# Exploring the Therapeutic Potential of Cannabinoid Receptor Antagonists in Inflammation, Diabetes Mellitus, and Obesity

**DOI:** 10.3390/biomedicines11061667

**Published:** 2023-06-08

**Authors:** Alexandru Vasincu, Răzvan-Nicolae Rusu, Daniela-Carmen Ababei, Monica Neamțu, Oana Dana Arcan, Ioana Macadan, Sorin Beșchea Chiriac, Walther Bild, Veronica Bild

**Affiliations:** 1 Department of Pharmacodynamics and Clinical Pharmacy, “Grigore T. Popa” University of Medicine and Pharmacy, 16 Universitatii Street, 700115 Iasi, Romania; dana.ababei@gmail.com (D.-C.A.); neamtu71@gmail.com (M.N.); radasanuoana@yahoo.com (O.D.A.); macadan.ioana@gmail.com (I.M.); veronica.bild@gmail.com (V.B.); 2 Department of Toxicology, “Ion Ionescu de la Brad” University of Life Sciences, 8 M. Sadoveanu Alley, 700489 Iasi, Romania; sbeschea@yahoo.com; 3 Department of Physiology, “Grigore T. Popa” University of Medicine and Pharmacy, 16 Universitatii Street, 700115 Iasi, Romania; waltherbild@gmail.com; 4 Center of Biomedical Research of the Romanian Academy, 700506 Iasi, Romania; 5 Center for Advanced Research and Development in Experimental Medicine (CEMEX), “Grigore T. Popa” University of Medicine and Pharmacy, 16 Universitatii Street, 700115 Iasi, Romania

**Keywords:** endocannabinoid system, diabetes, obesity, inflammation, immunomodulation, drug addiction

## Abstract

Recently, research has greatly expanded the knowledge of the endocannabinoid system (ECS) and its involvement in several therapeutic applications. Cannabinoid receptors (CBRs) are present in nearly every mammalian tissue, performing a vital role in different physiological processes (neuronal development, immune modulation, energy homeostasis). The ECS has an essential role in metabolic control and lipid signaling, making it a potential target for managing conditions such as obesity and diabetes. Its malfunction is closely linked to these pathological conditions. Additionally, the immunomodulatory function of the ECS presents a promising avenue for developing new treatments for various types of acute and chronic inflammatory conditions. Preclinical investigations using peripherally restricted CBR antagonists that do not cross the BBB have shown promise for the treatment of obesity and metabolic diseases, highlighting the importance of continuing efforts to discover novel molecules with superior safety profiles. The purpose of this review is to examine the roles of CB1R and CB2Rs, as well as their antagonists, in relation to the above-mentioned disorders.

## 1. Introduction

According to the World Health Organization (WHO), diabetes mellitus (DM) and obesity are considered epidemics due to their increasing incidence [1]. Obesity is involved in the etiopathogenesis of DM, as well as its complications, posing a significant threat to public health as an important determinant of insulin resistance [1]. The link between the two is well known and has even led to the development of the term “diabesity”, which is used to suggest the combined adverse health effects of the conditions [2]. Obesity affects both developed and developing countries, with alarming increases in prevalence rates [3]. Current estimates suggest that over 2 billion people have elevated body weight, and 641 million are obese [4]. The economic burden of obesity is also significant, with the annual expenditure on treatment in the United States (US) alone exceeding USD 211 billion [5].

In regard to DM, the WHO considers it a priority among non-communicable diseases (NCDs), and, alongside others, such as cardiovascular disease, cancer, and respiratory disease, DM accounts for the majority of premature deaths due to NCDs [6].

Although NCDs mainly fall into five disease groups (cancer, cardiovascular diseases, mental health, chronic obstructive pulmonary disease, and diabetes), diabetes is the only one that demonstrated a substantial increase in burden from 1990 to 2019 when evaluated by disability-adjusted life-years (DALYs) [7], being an important cause of DALYs: it was estimated that in 2017, it accounted for 2.6% of global DALYs [8]. According to the International Diabetes Federation (IDF), in 2017, there were approximately 451 million adults living with the disease, a number that is expected to grow to 693 million by 2045 if the current trend continues, while the 10th edition of the IDF Diabetes Atlas reports that, in 2021, more than 1 out of 10 adults were diabetic, and this number is expected to rapidly expand. In regard to costs, it is estimated that health expenditures related to diabetes were USD 966 billion in 2021. Costs are expected to increase to USD 1054 billion by 2045 [9,10].

An important feature of both obesity and DM is the inflammation of tissues [11]. This can be seen in pancreatic islets, muscles, the liver, and adipose tissue, all of which are insulin target tissues. Several studies have linked chronic tissue inflammation to these disorders, with tumor necrosis factor-α (TNF-α) levels being increased in adipose tissue in obesity. Proinflammatory cytokines can impair the action of insulin via the activation of signaling molecules (e.g., inhibitor of nuclear factor kappa-B kinase subunit β (IKK-β) and c-Jun N-terminal kinase (JNK)). Another determinant of insulin resistance is the increased number of adipose tissue macrophages (ATMs), which are the most abundant immune cells in adipose tissue [12]. Acute inflammation is frequently addressed with non-steroidal anti-inflammatory drugs (NSAIDs), as well as corticosteroids, but the long-term use of these medications can result in complications, such as gastrointestinal side effects and glucocorticoid-induced osteoporosis, making their use in chronic inflammatory diseases difficult [13,14]. Disease-modifying drugs such as methotrexate and leflunomide are useful in treating autoimmune diseases, but their effectiveness may decrease with continued use [15].

The peripheral inflammatory and immune responses in autoimmune diseases, such as rheumatoid arthritis, psoriasis, and Crohn’s disease, have been successfully modulated by biological drugs such as infliximab and adalimumab in recent years [16]. These drugs are largely ineffective in treating CNS inflammation and immune system irregularities. The blood–brain barrier (BBB) permeability prevents 98% of small-molecule medicines and all high-molecular-weight drugs from crossing [17]. In addition, neuroinflammation accelerates the progression of CNS disorders such as Alzheimer’s, Parkinson’s, and traumatic brain injury [18,19]. Hence, there is a major need for innovative treatment techniques to modulate inflammation, as well as neuroinflammation.

The ECS is a complex system that is involved in several physiological and pathological processes in mammals [20]. It is pivotal in the regulation of appetite, metabolism, and reward, with calorie intake and energy expenditure being some of the metabolic processes that it influences [21]. The major components of the ECS are its receptors and their endogenous ligands. These receptors are named cannabinoid receptors, with type 1 (CB1R) and type 2 (CB2R) being the most known, while anandamide (AEA) and 2-arachidonoylglycerol (2-AG) are their most studied endogenous ligands [22]. CBR can be found in several tissues, including the liver, brain, adipose tissue, and pancreas, and their activation leads to an increase in lipogenesis and decreased insulin sensitivity. It has also been suggested that marijuana, or *Cannabis sativa* (family *Cannabaceae*), may also regulate hunger, commonly referred to as “the munchies” [23]. ∆^9^-Tetrahydrocannabinol (∆^9^-THC), its main orexigenic component, increases energy expenditure [24].

Despite the success of existing treatments, obesity and diabetes remain major health concerns, and there is a continuous search for novel treatments that can better aid patients is of high importance, due to the multifactorial and complex nature of these disorders, as well as their complications.

Modulating this system with CBR antagonists has been shown to lower appetite and food intake while also enhancing insulin sensitivity and glucose metabolism. This has attracted interest in their potential involvement in the management of diabetes, obesity, the underlying inflammation, and their associated health conditions [25,26,27].

In spite of promising preclinical data, the development of such antagonists for clinical use has been hampered by their central side effects, which include psychiatric adverse events and an increased risk of depression and suicidal ideation. However, it is important to note that not all individuals experience these side effects, and the degree of their severity can vary widely due to interindividual reactivity. There are still numerous compelling reasons to continue and deepen the scientific study of their prospective usage. Ongoing efforts to develop new compounds with improved safety profiles hold great potential for the future, with peripherally restricted CBR antagonists that do not cross the BBB having shown promise in preclinical studies for the treatment of obesity and metabolic disorders.

The present review aims to explore the roles of CB1R and CB2R and their antagonists, with reference to several diseases, including DM, obesity, inflammation, and immunomodulation. Due to the fact there is still a stigma associated with cannabis use, and many people believe that all cannabinoids are harmful and addictive, this paper can also help to dispel myths and misconceptions about these compounds by providing accurate and evidence-based information and thus a more nuanced understanding of these compounds and their potential therapeutic benefits.

## 2. The Endocannabinoid System

The ECS plays a significant part in the regulation of energy balance, neurodevelopment, and other aspects of the body that show its involvement in the pathogenesis of diseases, as well as the prevention and treatment of illness [28]. The regulation of energy metabolism is greatly influenced by cannabinoid-receptor type 1 (CB1R) and type 2 (CB2R), their best-studied endogenous ligands, and enzymes for ECB synthesis and metabolism [26,27]. The best-studied primary endogenous ligands are AEA and 2-AG, which are synthesized from omega-6 polyunsaturated fatty acid (PUFA) and arachidonic acid (AA), respectively [29]. While AEA is a partial agonist with a high affinity for CB1Rs, 2-AG is a full agonist of CB1Rs and CB2Rs [30]. In addition to interacting with CBRs, both AEA and 2-AG can bind and activate the transient receptor potential vanilloid 1 (TRPV1). AEA is an agonist for several forms of the peroxisome proliferator-activated receptor (PPAR) family [31]. 

Aside from AEA and 2-AG, the body contains N-acyl ethanolamine and 2-acylglycerol, two other endogenous cannabinoids derived from n-6 and n-3 PUFAs such as palmitic, stearic, and oleic acids. These compounds can activate PPARs and other receptors, resulting in anti-inflammatory and appetite-suppressing biological activities [32].

AEA and 2-AG play a significant role in human brain homeostasis. Both are endogenously synthesized in the phospholipid bilayer and are degraded by related enzymes after interacting with membrane CBRs [33]. The biosynthetic enzymes for 2-AG are concentrated in post-synaptic neurons in dendritic spines and somato-dendritic compartments, despite the fact that the 2-AG concentration is substantially larger than that of AEA, showing its dominance in central homeostasis [28].

Neurotransmitter release is suppressed when the produced 2-AG binds to CB1Rs on the corresponding presynaptic neuron, blocking voltage-activated Ca^2+^ channels and boosting inwardly rectifying K^+^ channels [34]. Monoacylglycerol lipase then breaks down the 2-AG that was produced [33].

However, it has been hypothesized that AEA is presynaptically generated and that it reflects the levels of Ca^2+^ at axon terminals after they have been replenished from intracellular stores. AEA is taken up by post-synaptic cells after binding to presynaptic CB1Rs via transport proteins on neurons and glia that mediate endocannabinoid uptake [35,36]. Once within the cell, AEA is degraded by fatty acid amide hydrolase (FAAH), an enzyme found in the endoplasmic reticulum and attached to the cell membrane. It is not yet known how 2-AG is triggered to cross the plasma membrane of post-synaptic cells and interact with CB1 in the presynaptic milieu, but it is possible that passive carrier proteins are involved or that simple diffusion is responsible [36,37].

The two cannabinoid receptors (CBRs) share the common structural features of G-protein-coupled receptors (GPCRs), including seven hydrophobic domains that traverse the cell membrane, three extracellular and three intracellular loops, an extracellular N-terminal domain, and an intracellular C-terminal domain [38]. These receptors have the ability to modulate neuronal plasticity and are considered retrograde neuromodulators. Moreover, they can promote food intake, and under conditions of low energy, such as a fasting period, they cause energy accumulation. Essentially, lipid neuromediators known as ECBs operate in the hypothalamus by activating the sympathetic nervous system, thus signaling the body of low energy levels [39,40].

This biological system is involved in various homeostatic processes in the body, including appetite regulation. In addition to regulating body weight, it controls metabolism across the entire range from the CNS to the peripheral organs involved in digestion and energy storage [41].

As is well known, the CNS is responsible for processing sensory information and assessing the body’s energy needs. The sensation of hunger is mediated by the hypothalamus and is triggered by a series of hormonal imbalances, such as increased ghrelin and decreased circulating leptin, as well as the binding of 2-AG and AEA to hypothalamic CB1Rs [30].

CBRs are present in almost all tissues of mammals and are involved in various physiological processes. These processes include neuronal development, food intake, energy balance, and the modulation of the immune system. CB1Rs regulate neurotransmission and various peripheral functions, such as appetite modulation and homeostasis, while CB2Rs regulate immune and inflammatory pathways [42]. Antagonists of these receptors may aid in the treatment of obesity. Although different classes of CBRs share similar sequences, they respond specifically to the action of different compounds, such as phytocannabinoids, endocannabinoids, and synthetic cannabimimetic compounds [43,44].

### 2.1. Involvement of G-Protein-Coupled Receptors in CBR Activation

GPCRs are a group of receptors located on the surface of cells that play a critical role in the transmission of information from the cell’s exterior to its interior. This information transfer is mediated by heterotrimeric guanine nucleotide regulatory proteins (G proteins), which relay signals to other proteins, such as kinases. CBRs belong to the “class A” family of GPCRs, which share a common membrane topology consisting of an extracellular N-terminus, an intracellular C-terminus, and seven transmembrane helices connected by loops [45]. Key regions responsible for ligand binding, receptor activation, and signal transduction are situated within these seven transmembrane helices [46].

In general, ligands bind to specific sites in proteins, such as transmembrane regions, extracellular loops, or a combination of loops and residues in the binding site. Upon binding, the conformation of the protein changes, triggering the activation of G proteins that subsequently initiate a range of specific cellular responses [47].

Depending on the nature of their interaction, GPCR ligands can act as agonists, antagonists, partial agonists, or inverse agonists. Agonists initiate a cellular response by binding to the receptor and causing a conformational change. Antagonists bind to the receptor and prevent agonists from binding, resulting in no cellular response. Partial agonists, after binding, do not produce the complete conformational change of the agonist but still allow partial agonist activity. Instead, they partially activate the receptor and “block” it, reducing its availability for full-agonist binding. In the presence of both a full agonist and a partial agonist, the partial agonist acts as a competitive antagonist, resulting in a net decrease in receptor activation. Inverse agonists bind to a receptor and produce the opposite physiological response that an agonist would. Research has suggested that ligand-induced receptor activation typically occurs by altering the orientations of transmembrane domains 3 and 6 of the receptors [48].

The CNS plays a crucial role in regulating the delivery of nutrients to various tissues through the modulation of metabolic and molecular mechanisms. Energy homeostasis relies on the intricate coordination between homeostatic and nonhomeostatic reward circuits. Throughout the different stages of eating, spanning before, during, and after meals, peripheral signals originating from nutrients are transmitted to specific brain regions, notably the hypothalamic nuclei and the brainstem. These regions play a crucial role in regulating energy balance. Within them, orexigenic centers stimulate appetite, while anorexigenic centers suppress it, and they integrate these signals and generate appropriate responses to uphold metabolic and energy homeostasis in peripheral organs [49]. Furthermore, the act of feeding encompasses hedonic behavior, in which food is eaten for its own sake rather than to satisfy physiological needs. This nonhomeostatic regulation of energy balance stems from the rewarding properties associated with food, independent of nutritional needs. It involves the activation of specific brain regions, including the mesolimbic reward system, which encompasses the *ventral tegmental area* (VTA) and the *nucleus accumbens* (NAc) [50]. Additionally, the opioid, ECB, and dopamine systems contribute to this regulation. Collectively, these systems govern the aspects of food reward and the hedonic experience of eating [51].

ECBs play a crucial role in connecting dietary lipids to synaptic activity and neuronal plasticity, as well as neuroendocrine and reproductive functions. This emphasizes the significance of using proper dietary lipids to safeguard and uphold the specific molecular systems and mechanisms responsible for regulating neuronal functions, as well as preventing or treating brain disorders. An interesting example is the recognition of the “anti-aging” effects of dietary n-3 polyunsaturated fatty acids (PUFAs), which support cognitive processes and uphold synaptic functions and plasticity [52]. Conversely, diets rich in saturated fats have a detrimental impact on brain functions and increase the risk of cardiovascular and neurological diseases [53].

GPCRs exhibit a similarity of around 44% in terms of amino acid composition and a 68% homology in transmembrane domains when compared to human CB1Rs and CB2Rs. However, there are notable distinctions in specific regions between CB1 and CB2, including the N-terminal extracellular loop II (ECL2), which plays a significant role in cannabinoid binding, the C-terminal sequence of transmembrane domain 7, and the internal C-terminus [54].

The activation of CB1R triggers several intracellular pathways, such as the mitogen-activated protein kinase (MAPK) pathway, through which a cellular physiological response is initiated. The CB1R associates with Gq and Gs proteins in certain cell types. CB1R couples to Gs but with low efficacy compared to Gi/o [55].

Domains of the CB1R that selectively interact with Gi/o proteins have been identified and appear to be coupled to Gi/0α proteins in the absence of exogenous agonists [56]. CB1R elicits its physiological responses after coupling primarily to Gi/o proteins in order to inhibit adenylate cyclase and cyclic AMP signaling [57]. The CB1R-mediated inhibition of adenylyl cyclase activity was reported in neural and peripheral tissues, as well as in cells overexpressing CB1R [58].

Thus, the regulatory action of CB1 may encompass multiple aspects, such as ionic manifestations, as well as the activation of kinases. Calcium and potassium channels are opened in response to neuronal stimulation via CB1R.

The action of neuronal stimulation through CB1R results in the opening of calcium and potassium channels, leading to hyperpolarization and prolonged cellular effects, respectively, due to variations in the activity of transcription elements caused by the expression of multiple kinases. This will result in the regulation of multiple cellular actions, including protein synthesis [59]. The activation of CBRs will decrease cAMP levels and inhibit adenylate cyclase (AC) in most tissues because of their preferential coupling. Extracellular signal-regulated kinases-1 and -2 (ERK1/2), p38 MAPK, and JNK are all MAPKs that are regulated by both CBRs through phosphorylation and activation. Positive coupling to A-type K^+^ channels and negative coupling to N- and P/Q-voltage-gated Ca^2+^ channels are two additional functions of CB1Rs. CB1R can simultaneously activate G protein-dependent phospholipase C-b (PLC-b) to increase intracellular Ca^2+^ concentration [60].

### 2.2. Cannabinoid-Receptor Type 1

Although CB1Rs can be found throughout CNS, they play a particularly important role in regulating neuronal activity in the amygdala, hippocampus, cortex, and cerebellum. When stimulated, they block the neurotransmitters GABA and glutamate from being released. In addition, CB1R modulates the release of neurotransmitters in a dose-dependent manner by increasing the activity of potassium and calcium ion channels through the activation of the CB1R. The receptor can form both homodimer and hetero-oligomer complexes with other GPCRs, and it also has a binding site for allosteric modulators [47].

The adrenal gland, bone marrow, heart, lungs, and prostate are among the peripheral tissues that may express CB1R [61].

Multiple CB1R inhibitors have been found to date, and some are still in clinical phases of development. Preclinical studies investigating the neurological actions of obesity use CB1 blockers and compare their effects to those of other molecules. It is known that hunger is mediated by the hypothalamus and influenced by high levels of hormones such as ghrelin and low circulating levels of leptin. At the same time, a major role is played by the binding of two ECBs, 2-AG and AEA, to CB1Rs located in the hypothalamus. In obesity, the hyperactivity of the ECS has been observed, manifested by increased levels of AEA, which will lead to appetite stimulation by stimulating CB1Rs and, consequently, to weight gain. Another important issue is the involvement of the peripheral nervous system in the modulation of metabolism and digestion. CB1Rs can also be located at the gastrointestinal level, and by binding ECBs, gastrointestinal motility and the intensification of vasodilatation and inflammation can be induced. These receptors are also located on the insulin-producing β-cells of the islets of Langerhans. It is thought that ECB binding to these receptors will block the action of insulin because a heterodimeric complex is formed with insulin receptors [62].

The involvement of CBRs in fat storage should also be considered. Adipose tissue is made up of three types of cells: white, brown, and beige. White adipocytes are involved in fat storage and hormone release, brown ones are metabolically active, and beige ones can turn into white or brown cells. White adipose tissue stores carbohydrates and fatty acids as triglycerides and degrades them, releasing fatty acids into the bloodstream, in processes known as lipogenesis and lipolysis, respectively. When there is a surplus of food, adipocytes store it as triglycerides; when there is a shortage of food, the body’s lipid reserves are mobilized and reach other organs, such as the liver, muscle, and brown adipose tissue. Brown adipocytes have a lot of mitochondria and help keep the body’s temperature steady through thermogenesis, which uses fatty acids to make heat. Therefore, the activation of ECBs in white adipocytes inhibits thermogenesis. Blocking CB1Rs with pharmacological agents could induce their transformation into beige or brown adipocytes [63].

It was also proven that the activation of CB1Rs can cause skeletal muscle to decrease glucose uptake or the pancreas to release insulin [64].

### 2.3. Cannabinoid-Receptor Type 2

CB2R is expressed in the periphery, mainly in immune tissues, suggesting that ECBs have an immunomodulatory role [65,66].

CB2R is similar to CB1R in that it has seven transmembrane helices, a glycosylated N-terminus, and a C-terminal helix. It is mostly found in cells of the immune system, astrocytes, and microglia in the CNS. The activation of CB2R is linked to neuroprotective functions, and it also ensures the preservation of bone mass and a reduction in inflammation [26].

Recent evidence indicates that CB2Rs are also expressed in the brain and involved in neuropsychiatric functions even though they occur at much lower levels compared to CB1Rs. Furthermore, it has been shown that only the chronic activation of CB2Rs leads to increased excitatory synaptic transmission, while its short-term activation shows little effect on this activity. The use of the CB2 pathway as a target in obesity therapy would imply chronic neuronal activation, which, in turn, by increasing excitatory synaptic transmission, would facilitate peripheral anti-obesity effects. This effect would ideally not involve the induction of important psychotropic activity [27].

Immune signaling and inflammation are also regulated by CB2Rs. They are expressed on immune system cells and are frequently referred to as “peripheral” cannabinoid receptors. They are present in B- and T-cells, monocytes, macrophages, natural killer (NK) cells, and polymorphonuclear cells, as well as in the pancreas and lymphoid tissues, such as the thymus, tonsils, bone marrow, and spleen [67]. According to research, CB2 is most highly expressed by B-cells, NK cells, macrophages, and T-cells [68].

A small subpopulation of brainstem neurons also express CB2Rs, but little is known about the function of this cannabinoid receptor subtype in these cells. CB2 expression may also manifest in the CNS in response to an infection, an inflammatory condition, or times of stress. Still, the presence of these receptors at the CNS level is unclear, and more detailed studies are needed [69].

Chronic inflammatory conditions such as arthritis, autoimmune diseases, multiple sclerosis, HIV infection, stroke, and inflammatory bowel disease are linked to CB2R activation.

The effectiveness of CB1R agonists is limited by their short therapeutic window and psychoactive effects. On the other hand, CB2R agonists do not have psychoactive effects. Since neuroinflammation is a major cause of neurodegeneration, CB2R stimulation is a promising neuroprotective target. CB2Rs are found on leukocytes, which are involved in the anti-inflammatory and immune-modulating effects of cannabinoids [70]. This shows how they could be used to treat neuroinflammatory diseases. CB2Rs, on the other hand, have few CNS locations, which may explain their lack of side effects. CB2R activation may explain recent cannabis-related brain injury controversies, such as intracerebral hemorrhage [71].

Cannabinoids stimulate CB2Rs, and by inhibiting the activity of other chemoattractants, they may be able to reduce inflammation [72]. According to research, cannabinoids are extremely helpful in inflammatory states because they prevent the chemokine-induced chemotaxis of neutrophils, lymphocytes, macrophages, monocytes, and microglia [73].

In the treatment of inflammatory diseases, the administration of CB2R agonists may have an anti-inflammatory effect. Few CB2R agonists have been described in the literature up to this point, and even a patent review revealed that numerous CB2R modulators are currently in various stages of clinical development [74].

### 2.4. Signaling Process

CBRs lead to the induction of the MAPK cascade via the Gi/o protein when stimulated. CB1Rs and CB2Rs share only 48% amino acid sequence identity and differ in their sensitivity to agonists and antagonists. The activation of CBRs can lead to AC inhibition (which stops the conversion of ATP to cAMP) and the activation of MAPKs [75].

ECBs bind to CBRs in an autocrine manner. By activating CB1Rs in the brain, ECBs function as reversible antagonists of neurotransmitter secretion. The stimulus leading to post-synaptic ECB production is linked to increased Ca^2+^ levels due to the upregulation of ionotropic receptors. During autocrine stimulation, CB1Rs are activated by endocannabinoids that diffuse across the plasma membrane [25].

**Figure 1:**
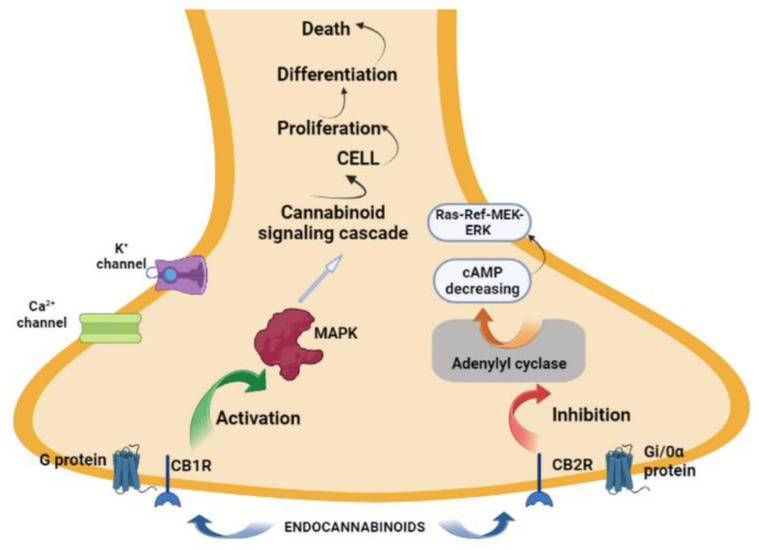
Involvement of cannabinoid signaling cascades in regulation of physiological mechanisms. Legend: cAMP: cyclic adenosine monophosphate; MAPK; Mitogen-activated protein kinase; MEK: Mitogen-activated protein kinase kinase; ERK: Extracellular signal-regulated.

G-protein coupling has an impact on the signal transduction mechanisms of CBRs, which belong to the rhodopsin subfamily of GPCRs. They are known to be activated by mechanisms including AC regulation, modifications in MAPKs, the modulation of ion channels, and variations in intracellular Ca^2+^. Cannabinoid signaling cascades are key factors in the processes of cell proliferation, cell differentiation, and cell death. Therefore, it is of significant importance to understand the process of CBR signaling in physiological mechanisms (Figure 1) [76].

Some GPCRs regulate cellular effectors, such as ion channels, enzymes, and GTPases, in response to stimuli. When it comes to coupling cell surface receptors to intercellular effectors, Go is by far the most prevalent G-protein in the CNS. The ability to detect, decode, and react to signals from outside the cell is crucial [77].

CB2Rs influence the Ras-Raf-MEK-ERK (MEK: mitogen-activated protein kinase kinase) pathway, which has an impact on mature tissues, by inhibiting AC activity via their Gi/Goa subunits and coupling to stimulatory Gi/o subunits [78]. The MAPK/ERK pathway is another name for the intracellular signaling cascade that connects DNA in the cell nucleus to the cell surface. In MAPKs, the Ras/Raf/MEK/ERK cascade reaction is a crucial signaling pathway. The corresponding cell surface receptors can be activated by a variety of stimuli, which then activate the signal transduction pathway and cause the proper biological response. Raf is recruited and activated by Ras, and later, Raf serine/threonine protein kinase promotes the dual-specificity protein kinase MEK1/2 (MAPK/ERK kinase) and the activation of ERK1/2. Different transcription factors and substrates are phosphorylated by the activated ERK1/2, which ultimately results in distinct patterns of gene expression [79].

## 3. The Endocannabinoid System in Inflammation and Immunomodulation

### 3.1. Modulation of Endocannabinoid System and Inflammation and Immune Process

Therapeutic applications for the treatment of cancer, arthritis, addiction, neuroprotection, pain, and inflammation may be possible through CB2R modulation, an intriguing strategy that lacks the typical CB1R-related psychotropic adverse effects [74].

Controlling the movement of inflammatory cells toward the site of injury is a promising avenue for developing new immunomodulatory treatments for various types of acute and chronic inflammatory conditions [80]. Among different available therapeutic options, the modulation of the endocannabinoid system has garnered significant interest.

Immune system cells such as leukocytes, neutrophils, monocytes, and T-cells have been shown to contain CB2Rs, which play a role in regulating the onset and progression of acute inflammation [81]. Compared to NK cells, monocytes, polymorphonuclear neutrophils, CD8+ and CD4+ T-cells, and B-cells fare better [73]. Since CB2Rs are highly concentrated in immune cells and their expression can be induced by proinflammatory stimuli, it is reasonable to assume that CB2R signal transduction plays a significant role in mediating immune and inflammatory responses [82].

### 3.2. Cannabinoid Receptor Modulators in Inflammation and Immune Processes

By interacting with CBRs on cell membranes, cannabinoids have also been shown to modulate the immune system, being considered a potential therapeutic target for peripheral and central inflammatory diseases [73,83].

The constitutive activity of CB2Rs paves the way for the development of inverse agonists, which provide an alternative mechanism for regulating CB2 action [84].

In different models of pain (acute, chronic inflammatory, post-operative pain, cancer, neuropathic), CB2R agonists demonstrated anti-nociceptive effects [85,86]. By contrast, it has been reported that inverse agonists/antagonists of CB2R are effective anti-inflammatory drugs [87].

According to Thomas et al., cannabidiol (CBD) is a highly potent non-competitive antagonist of both CB1Rs and CB2R in vitro. One possible explanation for its anti-inflammatory effects is that it induces CB2R inverse agonism at concentrations below those at which it displaces [^3^H] CP55940 from these receptors [88].

The ECS has been linked to its immunomodulatory function via the CB2 pathway [89]. The induction of regulatory T-cells and apoptosis, the inhibition of their proliferation, and the suppression of proinflammatory cytokines could explain its immunosuppressive effect [85,86,90]. Multiple studies have found that CB2 pathway modulators can have a beneficial effect on the equilibrium between the expression profiles of proinflammatory Th1 cytokines and anti-inflammatory Th2 cytokines [91]. The inhibition of AC and cyclic AMP formation by Gi signaling has been linked to CB2R activation and immune regulation [92]. It has also been shown that the MAPK pathway, which includes ERK, p38, and JNK, is under the control of the CB2 pathway [93].

Cell proliferation, as measured by the ^3^H-thymidine incorporation method, was not affected by the CB2R inverse agonists SR144528 and AM630, as shown by the work of Feng et al. These effects resulted from the control of important transcriptional factors, such as Bcl-6 and Pax5, by the protein kinase STAT3. According to the ^3^H-thymidine incorporation method, the CB2R inverse agonists SR144528 and AM630 inhibited IL-6-induced IgM production in human SKW 6.4 cells but had no effect on cell proliferation. The protein kinase STAT3 pathway and important transcription factors, such as Bcl-6 and Pax5, were responsible for these features. Furthermore, pretreatment with a CB2R agonist also prevented SR144528 from inhibiting IL-6-induced IgM secretion [94].

It has been discovered that certain molecules can selectively target CB2R, demonstrating only anti-inflammatory effects. CB2 signaling has been shown to impede disease progression in a variety of inflammation models, including experimental autoimmune encephalomyelitis (EAE), spinal cord injury, and stress-induced neuroinflammation, by decreasing the production of proinflammatory cytokines and blocking the recruitment of leukocytes to the site of inflammation [66].

Iwamura et al. found that the inverse agonists/antagonists JTE-907 and SR144528 prevented carrageenan-induced paw edema in mice [83]. CB2Rs have been linked to the development of edema. In addition, Rinaldi-Carmona et al. further investigated SR144528 and found a possible in vitro mechanism of action. Researchers discovered that the compound could counteract CP 55,940’s inhibition of forskolin-stimulated AC activity in CHO-CB1 cells expressing CB2Rs [95]. In addition, SR144528 was able to selectively inhibit CP 55,940-induced p22/p44 MAPK activity in cells expressing CB2Rs. Human tonsillar B-cell activation induced by Ig cross-linking was also inhibited by SR144528, while CP 55,940 had no effect [91].

Using human embryonic kidney 293 (HEK293) cells stably expressing CB2R, Kumar et al. demonstrated that SR144528 increased forskolin-stimulated cAMP accumulation. Based on a comprehensive screen of 640 compounds approved by the FDA as potential ligands to CB2R, the researchers found that the selective estrogen receptor modulator raloxifene exhibited CB2R behavior similar to that of SR144528. It has also been reported that raloxifene acts as a competitive antagonist to the effects of the synthetic cannabinoid agonists CP-55,940, HU-210, and WIN55,212-2. These results establish the feasibility of using this drug, which targets CB2R, in new therapeutic contexts [96].

In a mouse model of irritable bowel disease (IBD), Gentili et al. demonstrated the anti-inflammatory effect of JTE-907 [97]. The compound had no effect on the development of T-cell subtypes such as Th1, Th2, Th9, or Th17 but activated the p38 MAPK pathway, which is associated with suppressive activity and the differentiation of naïve peripheral CD4+CD25- T-cells into suppressor-induced Treg cells [98]. Inflammatory and autoimmune disorders are now widely known to be associated with Treg cell dysfunctions. Therefore, a way of treating the above-mentioned diseases through the pharmacological modulation of these cells is of great interest [99].

The findings of Ueda et al. revealed that repeated topical treatment with dinitrofluorobenzene (DNFB) triggered allergic dermatitis in mice and that the CB2R antagonist/inverse agonists JTE-907 and SR144528 prevented this inflammation. Based on these results, it seems that CB2R is partly responsible for local inflammatory responses [100].

Particularly in macrophages and macrophage-like cells, as well as CNS-resident microglia, CB2R has been shown to play a functionally significant role during the inflammatory process [91]. Based on a 2,6-dihydroxy-biphenyl-arylmethanone scaffold, Alghamdi et al. developed the selective CB2R inverse agonist SMM-189. In several in vitro studies, it was found that SMM-189 inhibited microglia polarization toward the proinflammatory M1 phenotype and the pro-healing M2 phenotype [101]. Additionally, the compound suppresses the expression of inflammatory cytokines and chemokines in primary microglia derived from humans and mice [102,103]. 

In another study by Yu et al., SMM-189 reduced inflammatory responses in classically activated microglia and prevented neuronal damage due to glutamate excitotoxicity in primary cultures of brain cells isolated from rat hippocampal tissues. Otherwise, the treatment with SMM-189 only partially decreased inflammatory markers and diminished the severity of comorbid behavioral deficiencies in a mouse model of status epilepticus induced by kainite [87]. 

It is possible that protecting neurons from damage requires more than just targeting CB2R. This suggests that ECBs and the COX/prostanoid cascade signaling systems may interact during inflammatory processes triggered by external aggression to the brain. It was shown that SMN-189 treatment reduced the increased levels of COX-2 in the hippocampus and cerebral cortex, thereby reducing injury and functional deficits after prolonged seizures [104].

Sch. 414319 is a triaryl bis-sulfone that binds as an inverse agonist to CB2Rs. The clinical symptoms of EAE in the Lewis rat strain are reduced and cell migration in vivo is suppressed, as is bone destruction in antigen-induced mono-articular arthritis. As a mechanism separate from chemokine receptor desensitization, Lunn et al. proposed that controlling L-plastin phosphorylation is responsible for these effects on inflammatory cell migration [84].

Lunn et al. reported the pharmacological characterization of Sch.336, another CB2R inverse agonist/antagonist. The compound blocked CB2-expressing recombinant cells from moving toward the CBR agonist 2-AG in vitro. Sch.336 modified leukocyte migration in the presence of the CBR agonist HU-210 and chemokine-induced cell recruitment into a CCL2-soaked gel foam sponge in several in vivo experiments. The compound also inhibited antigen-induced pulmonary and peritoneal cell infiltration [105].

The maintenance of bone mass is heavily reliant on the endocannabinoid system, with both CB1Rs and CB2Rs expressed on human osteoclastic and osteoblastic cells. Experimental evidence suggests that CB2R antagonists may be effective as antiresorptive agents in the treatment of postmenopausal osteoporosis [106]. For instance, Geng et al. reported that 6-iodopravadoline (AM630) inhibited the expression of CB2R in RAW 264.7 cells and reduced inflammatory osteolysis caused by titanium particles generated due to prosthesis wear. In addition, treatment with 100 nM AM630 significantly decreased the protein expression of specific proinflammatory cytokines, namely, interleukin-1β (IL-1β) and TNF-α, in RAW cells cultured with titanium (Ti) particles, suggesting its potential as a therapeutic agent for the treatment of aseptic loosening associated with Ti particles [107].

Additional research has demonstrated that SR144528 can limit bone loss by inhibiting the formation of osteoclasts and reduced bone resorption [108]. These findings are consistent with those of Sugiura et al., who reported that SR144528 has an antagonistic effect against 2-AG-stimulated Ca^2+^ influx in macrophage-like cells differentiated from HL60, U937, and THP-1 cells and human peripheral blood monocytes [109]. A contradictory effect was observed for AM630, which was ineffective in the same assay, indicating that CB2R inverse agonists may have varying effects on the influx of Ca^2+^ triggered by 2-AG [110].

Another group of researchers demonstrated that the prior administration of AM630 activated the influx of Ca^2+^ into sensory trigeminal (TG) neurons, resulting in the pharmacological cross-desensitization of the TRPV1 channel. When co-expressed with TRPV1, it was shown that AM630 activated the transient receptor potential ankyrin 1 (TRPA1). The pretreatment of cultured TG neurons with the compound inhibited capsaicin-, WIN55,212-5-, and mustard-oil-induced responses, as was observed in the study [111].

Recent advances in RNA and protein detection, as well as genome-editing techniques, have shown that CB2Rs are also present in the brain across different species, dispelling the long-held belief that they are exclusively expressed in the periphery. Memory loss following surgery in mice was associated with elevated CB2R expression in the hippocampus and prefrontal cortex. This suggests a potential role for CB2Rs in cognitive deficits. These effects were attenuated by the CB2R antagonist AM630 and exacerbated by the CB2R agonist JWH133 [112]. Another study showed that the CB2R inverse agonist SMM-189 decreased neuronal death in the cortex, striatum, and amygdala following traumatic brain injury (TBI) in a mouse model. This was accompanied by a restoration of normal oscillatory activity in the hippocampus and the prefrontal cortex, both of which had been disrupted by the TBI [113].

Recent data from the literature regarding the effects of CBR modulators on inflammation and immunomodulation are presented in Table 1.

## 4. The Endocannabinoid System in Diabetes Mellitus

Diabetes mellitus (DM) is a disorder of carbohydrate metabolism involving increased glucose uptake with the development of chronic hyperglycemia, leading to the development of comorbidities that result in a decreased quality of life, high financial costs of care, and early mortality.

Although it comprises several subtypes (e.g., gestational diabetes, neonatal diabetes, maturity-onset diabetes of the young), the most known are type 1 (T1DM) and type 2 (T2DM) [114].

Of the two main types, T2DM is more common, representing more than 95% of all diabetes cases globally [115]. This is characterized by the inability of tissues that are sensitive to insulin to appropriately respond to its action as well as by modifications in insulin secretion by the responsible cells. Emergent pathophysiological factors involved in the disease include adipokine and immune dysregulation, inflammation, and abnormalities in gut microbiota [116].

T1DM constitutes about 5–10% of all diabetes cases and is an autoimmune disease in which pancreatic β-cells are destroyed, mediated through T-cells. Several immune markers, particularly autoantibodies, are associated with the destruction of β-cells, and this leads to insulin deficiency and finally to hyperglycemia [117].

In children and adolescents, the prevalence of both types of DM has increased. It is estimated that the number of patients under the age of 20 with T1DM is higher than 1 million [6], while around 41,600 new cases of children and adolescents were diagnosed with T2DM in 2021 globally [115].

Abnormal levels of blood glucose manifest as dysfunctions in the metabolism of carbohydrates, proteins, and fat. This metabolic disruption affects multiple organs, which can ultimately lead to organ failure, a state that characterizes diabetes complications. These complications affect different parts of the body, with an emphasis on the eyes, nerves, kidneys, and heart [117]. Cardiovascular, cerebrovascular, and peripheral vascular diseases are classified as macrovascular complications of diabetes, while retinopathies, nephropathies, and neuropathies are considered microvascular complications [118]. These associated complications contribute to the cost of diabetes, which is prevalent across all socioeconomic layers [119] and is an important cause of productivity loss due to morbidities and premature mortality [8,120]. The common denominator for most diabetic complications is represented by abnormalities in the vascular system [65].

Inflammation is one of the most important contributors to the development of diabetic complications, with cardiac inflammation being an early and common symptom of diabetes and playing a key role in diabetic cardiomyopathy, which contributes to the progression of heart failure [121]. 

According to the research of Jaesin et al., it appears that a significant number of patients experience complications related to DM when they are first diagnosed with the condition. Specifically, the study found that approximately 12% of individuals with T2DM already had chronic kidney disease at the time of diagnosis [122].

### 4.1. Complications of Diabetes Mellitus

Diabetic cardiomyopathy is a specific type of cardiomyopathy observed in diabetic patients without other cardiovascular disorders, characterized by myocardial hypertrophy, interstitial fibrosis, and microcirculatory abnormalities resulting from DM [123]. Hyperglycemia increases oxidative stress and reactive oxygen species, leading to DNA damage and apoptosis through proinflammatory and stress signaling pathways, causing myocardial cell death. Fibrosis, caused by an increase in collagen formation, results in reduced heart contractility [65]. According to biomarkers of cardiovascular risk, elevated plasma levels of ECBs have been associated with harmful coronary circulatory events in obese patients [124,125]. In a mouse model of T1DM cardiomyopathy, higher levels of AEA and CB1R expression were found in diabetic hearts, causing the accumulation of advanced glycation end products, oxidative/nitrative stress, cell death, inflammation, and fibrosis. These processes were improved and remodeling and diabetic cardiac dysfunction were reversed by CB1R blockade or genetic deletion. Additionally, in an angiotensin II-dependent hypertensive rat model, CB1R inhibition improved cardiac function and remodeling after myocardial infarction and metabolic syndrome, improving blood-pressure regulation and the metabolic profile [126,127,128].

Diabetic nephropathy (DN) is a complication of both T1DM and T2DM, characterized by structural changes and a decline in renal function [129]. Oxidative stress, inflammation, and fibrosis contribute to its formation and progression. Studies on animal models of DN have highlighted that the modulation of the ECS shows a renoprotective effect. In murine models of DN, CB1R antagonists such as rimonabant have reduced glomerular damage, prevented proteinuria, and improved renal function by improving the albumin–creatinine ratio and glomerulosclerosis in obese Zucker diabetic fatty (ZDF) rats. Rimonabant decreased urinary albumin excretion and mesangial expansion and suppressed the synthesis of profibrotic and proinflammatory cytokines, according to a study on a mouse model of T2DM [126]. CB1R blockade has also improved insulin resistance and lipid profiles, possibly explaining its renoprotective effect. Enhanced CB1R signaling promoted the generation of reactive oxygen species (ROS) in podocytes and cell death, while the blockade of peripheral CB1R with JD5037 prevented and reversed specific symptoms of diabetic nephropathy [130]. The use of CB1R antagonists such as AM-6545 has shown renoprotective potential by decreasing urinary microproteins, renal collagen, and hypertrophy as well and providing protection against albuminuria [131].

Diabetic peripheral neuropathy (DPN) is a common complication of T2DM that damages the peripheral and autonomic nervous systems, and it is caused by oxidative stress, poor insulin signaling in the brain, and inflammation, all of which are pathological consequences of obesity-related insulin resistance. The most common type is distal symmetric polyneuropathy, affecting the hands and lower limbs [132]. Patients often experience electric, burning, or stabbing pain. Studies have shown that peripherally restricted CB1R agonists can have an analgesic effect, but the inhibition of these receptors may also be beneficial. When using rimonabant in animals with streptozotocin (STZ)-induced diabetes, the perception threshold was increased, while the loss of intraepidermal nerve fiber density was partially prevented. Rimonabant has been shown to reduce mechanical allodynia as well as oxidative stress in peripheral nerves and to inhibit TNF-α overexpression in the spinal cord. Although further studies are required, it is evident that the modulation of the ECS could lead to favorable results in managing DPN [133].

One of the most common microvascular complications of DM that can cause permanent vision loss is diabetic retinopathy (DRP) [134]. CB1R activation can contribute to oxidative damage and inflammation, which can lead to cell death, but the precise role of the ECS in this complication is still unclear. CB1R antagonists, such as rimonabant and AM251, have been shown to reduce apoptosis and oxidative damage in vitro when applied to retinal pigment epithelial cells that have been exposed to high glucose [135]. Retinal endothelial cell death was prevented by blocking CB1R in both rodent and human models of DRP [136]. The CB1R antagonist SR1 decreased photoreceptor loss and glial activation in a mouse model of retinal degeneration induced by N-methyl-N-nitrosurea (MNU), indicating that the modulation of the ECS may offer effective treatment options for DRP [137].

**Figure 2:**
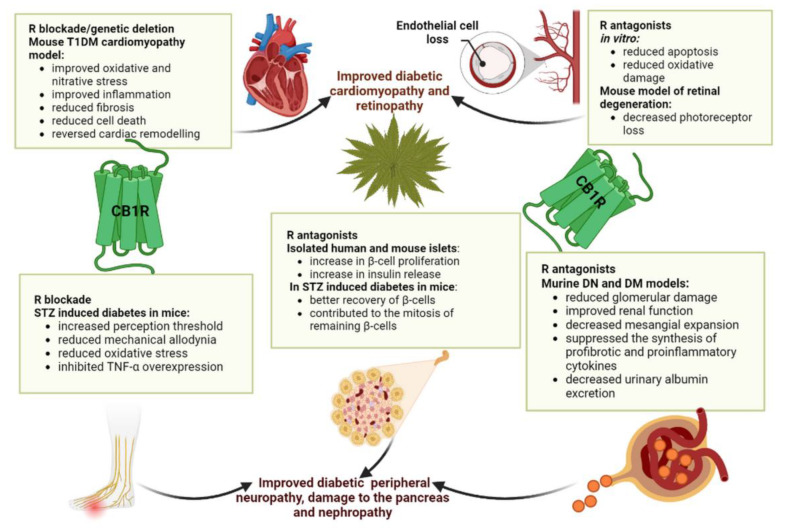
Involvement of CB1Rs in the complications of diabetes mellitus. Legend: R: receptor; T1DM: Type 1 diabetes mellitus; DM: Diabetes mellitus; DN: Diabetic nephropathy; STZ: streptozotocin; TNF-α: tumor necrosis factor-α.

In Figure 2, the main implications of CB1Rs in the complications of diabetes mellitus are presented.

### 4.2. Modulation of Endocannabinoid System and Diabetes Mellitus

The involvement of the endocrine pancreatic ECS in the regulation of metabolic homeostasis has been demonstrated by in vitro studies on mouse islets, which have shown that, physiologically, there are correlations between insulin secretion and CBRs [138,139]. It is associated with energy balance and insulin and glucagon secretions, contributing to glucose homeostasis and metabolism [140].

Through its actions, the ECS favors the accumulation of fat, and its overactivity can lead to the pathogenesis of obesity, which is an important cardiovascular risk factor for diabetic patients, with abdominal obesity being a key risk predictor for cardiovascular as well as metabolic diseases. Adipose tissue contributes to the release of inflammatory cytokines such as TNF-α, IL-6, other inflammatory markers (e.g., C-reactive protein), as well as hormones such as leptin and adiponectin. These cytokines are a cause of insulin resistance, leading to histological alterations in the adipose tissue and thus leading to insulin resistance: exacerbated inflammatory cytokines result in insulin-resistant adipocytes, insulin resistance in skeletal muscle as well as reduced insulin secretion from pancreatic cells, and a decrease in glucose production suppression in hepatic cells [141]. In a preclinical study of obesity, the activation of CB1R in hepatocytes and skeletal muscle was shown to be linked to systemic glucose intolerance as well as insulin resistance [142].

The higher gene expression and protein levels of FAAH observed in the livers of diabetic animals compared to control animals support the hypothesis that ECS activation and an increase in ECB synthesis also lead to an increase in the activity of their main degrading enzymes, possibly via a feedback mechanism. FAAH plays a role in both apoptotic and necrotic cell death in hepatocytes, in addition to its role in AEA degradation [143].

Preclinical studies in both types of DM have demonstrated that the ECS is involved, with evidence that the activation of CB2Rs could enhance responses by reducing the risk of stroke, as well as other neurological deficits. These brain manifestations are driven by the already-impaired reactivity of cerebral arterioles caused by increased metabolic demand. In T1DM, endothelial nitric oxide synthase (eNOS)- and neuronal nitric oxide synthase (nNOS)-dependent responses at the arteriolar level are altered. These benefits could be observed in a rat model of T1DM induced by streptozotocin administration. Subsequently, animals were treated with the CB2R agonist JWH-133, and the results of the study showed that acute therapy with this agonist could improve eNOS- and nNOS-dependent responses. According to these data, treatment with CB2R agonists could have therapeutic effects by preventing stroke [144].

An increase in hepatic TNF-α, apoptotic cells, IL-6, and FAAH gene and protein expression and the depletion of hepatic CB1R are all hallmarks of the liver damage caused by T1DM. Therefore, agents that can modulate ECS components, such as CB1R or the FAAH enzyme, may be useful in treating diabetes-related liver damage [143]. 

It also has been suggested by various studies that the overactivation of the ECS system contributes to insulin resistance in T2DM, along with dyslipidemia and obesity. These pathologies are believed to lead to glucose intolerance, reduced insulin sensitivity, and weight gain [145,146].

### 4.3. Cannabinoid Receptors in Diabetes Mellitus

It has recently been suggested that CBRs can be found in pancreatic Langerhans islets, specifically in β-cells, in humans, mice, and rats and that ECBs are also produced in these islets [62]. The activation of CB2R located on Langerhans islets is crucial for regulating glucose homeostasis and insulin secretion, both of which are related to ECB signaling in these islets [133,146,147]. More specifically, it appears that ECBs are generated in β-cells as a result of depolarization [62,148,149].

At the level of Langerhans islets, CB1Rs are predominantly found in α-glucagon-secreting cells, whereas CB2Rs are distributed among both α- and β-cells and play a role in the synthesis of insulin and glucagon. The alteration of insulin secretion seems to be influenced by the activation of CB1Rs and CB2Rs by ECBs or similar compounds, with the consequence of this activation being the result of oscillating changes in the calcium levels involved in insulin synthesis [150,151,152]. It seems that in patients with diabetes, the mass of β-cells is reduced, especially in T1DM, and CB1R negatively impacts the size of these cells.

While evidence points toward the fact that AEA and 2-AG decrease insulin secretion in β-cells and islets isolated from mice and rats, CB1R and CB2R activation via AEA or synthetic agonists potentiate glucose-stimulated insulin secretion (GSIS). However, these contradictory results could be due to the origin of cell cultures, the concentration of ligands, or experimental conditions, all of which could be reasons for disparities [140].

The activation of CB1Rs has been shown to decrease glucose uptake in skeletal muscle and insulin secretion from the pancreas, as stated by Orio et al. [64]. The activation of phosphatidylinositol 3-kinase (PI3K) and an increase in intracellular calcium levels, which mediate glucose uptake upon CB1R activation, may account for the insulin-mimetic effect of ECBs, according to Pagano et al. [153]. CB1R activation leads to the suppression of GSIS, whereas the absence of this receptor type is associated with enhanced GSIS.

CB1R activation contributes to systemic inflammation by increasing IL-6 and TNF-α expression. It leads to ROS formation as well as inflammation associated with diabetes, thus leading to the additional production of endocannabinoids, tissue injury, and well-known diabetic complications (e.g., neuropathy, cardiopathy, retinopathy, nephropathy) [154].

Modifications in β-cells due to inflammation and nutrient overload involve CB1R as well. The activity of other receptors (such as TRPV1) that affect intracellular Ca^2+^ levels can also be altered, as can the inhibition of insulin receptor autophosphorylation. These two mechanisms counteract the insulin secretagogue actions of incretins (glucagon-like peptide-1 and glucose-dependent insulinotropic polypeptide) [62,155,156]. GLP-1-mediated insulin secretion appears to be enhanced by this blockade in mice [155].

Pisanti et al. demonstrated ECS involvement in the angiogenic process, suggesting that the inactivation of CB1R leads to angiogenesis inhibition, which is of interest, considering the neovascularization processes associated with diabetic complications [157].

CB1R blockade in ZDF rats led to an increase in the gene expression of adiponectin, which was not seen in CB1-/- mice [158]. In addition, it increased serum adiponectin levels in diet-induced obese mice, which might explain the resistance to hyperinsulinemia and hyperglycemia induced by a high-fat diet in CB1-/- knockout mice [159,160].

However, other studies argue that obesity-related inflammation and insulin resistance are potentiated by CB2R, a receptor involved in regulating heightened immunity and the inflammatory response in different situations. Local inflammation is promoted by proinflammatory macrophages, which are recruited by adipocytes in adipose tissue and which, by producing cytokines, TNF-α, chemokines, and other factors, trigger the inflammatory response [142,161].

### 4.4. Cannabinoid Receptor Modulators in Diabetes Mellitus

Endogenous CB1R ligands, as well as synthetic agonists, can inhibit insulin secretion due to glucose stimulation via the inhibition of AC activity and cAMP formation. This reduces the movement of secretory granules that contain insulin to the surface of the cells. The opening of Ca^2+^ channels is also reduced, leading to the suppression of insulin secretion from β-cells [155].

A variety of CB1R antagonists have been shown to increase insulin release in mouse and rat cells as well as human islets [62]. The treatment of obese mice with a CB1R antagonist increased energy expenditure and insulin-stimulated glucose disposal by increasing glucose uptake and oxygen consumption in skeletal muscle [139].

Thus, it was observed that by exposing isolated human as well as mouse islets to CB1R antagonists (AM251 and SR141716A (rimonabant)), an increase in β-cell proliferation was induced, which was also seen in mice injected with AM251, which presented larger islets. AM251 contributed to the better recovery of β-cells as well as to the mitosis of the remaining β-cells in mice treated with streptozotocin, a β-cell toxin [154].

Myocardial oxidative and nitrative stress markers were reduced in doxorubicin-treated mice with the deletion of CB1R. Similarly, doxorubicin-induced oxidative and nitrative stress and related cell death were attenuated by CB1R blockers such as AM281 and rimonabant [65].

Rimonabant use led to a decrease in hemoglobin A1C values in the RIO-Diabetes trial in patients on treatment with either metformin or sulfonylurea. Rimonabant administered in a dose of 20 mg daily also led to a significant reduction in triglycerides (9.1%) while also increasing high-density lipoprotein (HDL) cholesterol levels (15.4%) in T2DM patients. A CB1R blocker also reduced the C-reactive protein (CRP) levels during a 1-year treatment in overweight or obese patients with dyslipidemia, independent of statin treatment [162,163]. This is important, since CRP, as a marker of a proinflammatory state, is predictive of cardiovascular disease [138].

By blocking CB1R, it was shown that obesity-induced insulin and leptin resistance, as well as glucose homeostasis, was improved in obese and overweight animals with metabolic syndrome. Thus, a 14-day intraperitoneal administration of rimonabant reduced body weight and food intake in non-obese chow-fed rats in a dose-dependent manner. It seems that at the central level, the mechanism behind the above-mentioned results was due to the enhancement of the satiety center as well as the inhibition of ghrelin. This led to the attenuation of the mesolimbic dopamine system. At the peripheral level, rimonabant acts by decreasing the levels of circulating ghrelin, as well as gastrointestinal motility, thus reducing the absorption of nutrients [154].

The inhibition of basal insulin hypersecretion due to obesity and the inhibition of glucose-stimulated insulin secretion were identified after the direct in vitro administration of rimonabant in islets [164].

Although promising at first, rimonabant was retracted from the pharmaceutical market because of the psychiatric disorders it caused, of which depression and an increase in suicidal thoughts are of most importance [154]. It is currently seen as a starting point for the development of other CB1R antagonists/inverse agonists that do not cross the blood–brain barrier and do not have its adverse profile. Such compounds target peripheral tissues and increase lipid mobilization and decrease triglyceride storage as well as glucose production, leading to weight loss, a reduction in food intake, an improved lipid profile, and ameliorated insulin intolerance. This could lead the way to the development of potent and safe management options for diabesity [142].

AM251 is a structural analog of rimonabant with a different halogen (I instead of Cl). It seems that its efficacy and potency are similar to those of rimonabant, while its binding affinity for CB1R is reported to be higher. AM251 was shown to reduce food intake in food-restricted rats and in two mouse models of obesity [165,166].

In a study that aimed to evaluate the sub-chronic efficacy of AM251 in young obese diabetic mice, it was shown that its administration led to a decrease in food intake and had a beneficial effect on glucose concentrations and overall glycemic excursion in ob/ob mice, which the authors explained by improved insulin sensitivity in peripheral tissues [167]. AM251 also has a direct beneficial effect on islet function by preventing apoptosis in isolated mouse and human islets and promoting insulin secretion from isolated human islets [168]. While it is a widely used research tool, its centrally mediated psychiatric side effects highlight the importance of developing peripherally restricted CB1R antagonists that lack this adverse profile [169].

Because of the risk of side effects, ongoing clinical trials for brain-penetrant CB1R antagonists, such as ibipinabant, taranabant, surinabant, and otenabant, were terminated [169].

AM6545 is a CB1R-specific neutral antagonist with limited penetration in the CNS without major side effects due to the fact that it cannot cross the blood–brain barrier because of its reduced liposolubility. AM4113 is a brain-penetrant CB1R neutral antagonist that can act both centrally and peripherally. It is more potent than rimonabant and lacks its central side effects [170].

Both AM6545 and AM4113 were shown by Eid et al. to reduce insulin levels and the insulin resistance index to near-control values. Both compounds prevented the development of insulin resistance in male Wistar rats fed a high-fructose/high-salt diet, suggesting that they are equally effective [171]. In regard to food intake, it was shown that both compounds reduced the body weight gain of animals. AM6545 inhibited food intake, resulting in lower weights in rats and mice [170], while AM4113 also reduced weight gain in rats by decreasing appetite [171]. The two compounds also led to the restoration of adiponectin levels in metabolic syndrome rats, suggesting their anti-inflammatory, anti-atherogenic, and anti-diabetic properties. Furthermore, the compounds led to a reduction in TNF-α to similar levels, an inflammatory cytokine that is increased in animal models of metabolic syndrome as well as diabetes [171]. 

In a study by Ma et al., AM6545 was administered to monosodium glutamate-induced obese mice with apparent glucose intolerance. The compound produced lower blood glucose levels at different times after glucose loading while also lowering fasting insulin levels. This indicates that the sub-chronic administration of AM6545 could improve insulin resistance in these animals [172].

Barutta et al. also showed that the blockade of CB1R along with ACE inhibition treatment can reverse albuminuria and nephron loss while also reducing inflammation in an animal model of T1DM with early diabetic nephropathy, results that could be of use for this complication in human patients [173].

JD5037 is a peripheral CB1R inverse agonist obtained by modifying the structure of its parent compound, SLV319, which penetrates the brain. JD5037 has limited brain penetrance and is known to be efficient in reducing body weight, food intake, hepatic steatosis, and insulin resistance. The compound also contributes to liver protection against fibrosis, as well as obesity-induced nephropathy [174].

In a study on STZ-induced diabetic mice, it was shown that JD5037, alongside its parent compound SLV319 (ibipinabant), normalized impaired kidney function, renal inflammation, and tubular injury after diabetes induction. Diabetes led to elevations in expression levels of collagen-3, collagen-1, and fibronectin-1, which were normalized by JD5037. Positive results for this compound were also observed in a genetic mouse model of diabetic nephropathy, in which inflammation, renal injury, tubulointerstitial fibrosis, and creatinine clearance were reduced [175].

TXX-522 is another highly selective CB1R antagonist with the potential to manage diabetes and associated metabolic disorders such as obesity. Wei Chen et al. found that TXX-522 and rimonabant both reduced fasting blood glucose and insulin levels in male Sprague-Dawley rats but that TXX-522 improved glucose intolerance more than rimonabant at the same dose, which was shown by oral glucose tolerance testing. While rimonabant was effective at reducing hypertriglyceridemia, a higher dose of TXX-522 was effective at reducing serum triglyceride and total cholesterol levels without causing any observable behavioral or other toxicity-related side effects (e.g., lethargy, mania). Accordingly, TXX-522 is hypothesized to have CB1R-selective in vivo antagonist activity with low brain permeability [176].

(S)-MRI-1867 is a CB1R/inducible nitric oxide synthase hybrid inhibitor with a limited peripheral distribution (iNOS). Roger et al. observed that in diet-induced obese mice, this compound led to gradual weight loss, which they attributed to a decrease in adiposity. This was only seen in obese mice. The levels of insulin and glucagon in the blood were lowered, and the blood sugar was lower when people were at rest. Glycemic control in the obese mice was improved, and leptin levels were lowered, while adiponectin concentrations remained unmodified. (S)-MRI-1867 also improved hepatic lipid metabolism, reduced dyslipidemia and de novo lipogenesis, and impaired cholesterol and lipoprotein anabolism [177].

AJ5012 is another peripheral CB1R antagonist that suppressed tissue inflammation through the nucleotide-binding domain and leucine-rich repeat protein 3 (NLRP3) inflammasome. This led to improved insulin sensitivity as well as glycemic control. Epididymal white adipose tissue in diet-induced obese mice showed decreased levels of the proinflammatory chemokine monocyte chemotactic protein 1, TNF-α, and interferon regulatory factor-5 (IRF5), a marker of inflammatory macrophage polarization. Proinflammatory gene expression was also confirmed to be induced by AEA in murine macrophage RAW264.7 cells. AJ5012 treatment inhibited CB1R, IRF5, NLRP3, and IL-1β, which was also seen in 3T3-L1 preadipocytes that differentiated into adipocytes. In the same manner, AJ5012 inhibited CB1R, TNF-α, IRF5, NLRP3, and IL1β induced by AEA administration. Therefore, AJ5012 was found to enhance metabolic parameters and insulin resistance via CB1R peripheral blockade. In both diet-induced obese and db/db mice, it reduced inflammation in adipose tissue by inhibiting the NLRP3 inflammasome [178].

Originally described as a silent CB1R antagonist with poor brain permeability, LH-21 is now known to be a peripheral CB1R neutral antagonist or weak inverse agonist. In genetic ZDF rats and diet-induced obese rodent models, it was found to reduce body weight and increase insulin sensitivity [179]. The effects of LH-21 on the pancreas and the liver were examined in a study by Zerbo et al. As a result of its cytoprotective effects on pancreatic islets and the liver in a mouse model of obesity and pre-diabetes, the authors drew the conclusion that it reduces diabetes risk factors, such as glucose handling and tissue inflammation. Reduced apoptosis and macrophage infiltration are the cause of this phenomenon. High-fat-diet-induced low-grade inflammation was also ameliorated by LH-21 [180]. Recent data from the literature regarding the effects of CBR modulators in DM are presented in Table 2.

## 5. The Endocannabinoid System in Obesity

Obesity is a complex disease related to metabolic dysfunction, characterized by hormonal dysfunction, impaired energy balance, and the irregular expansion of fat tissue, and is associated with increased mortality due to a high risk of developing comorbidities such as cardiovascular and liver-related disorders, DM, and others [43,44]. Moreover, maternal obesity and gestational diabetes are conditions that can cause multiple birth defects or even perinatal death worldwide [181].

### 5.1. Modulation of Endocannabinoid System and Obesity

A crucial factor in maintaining energy homeostasis is the ECS [182], with some authors describing it as a complex system (endocannabinoidome) with an essential role in metabolic control and in lipid signaling [183]. Its overactivation can lead to obesity, and the specific blockade of CB1Rs results in weight loss [44,181].

Human CB1R interacts with β-arrestin, which is involved in mediating receptor internalization. It is widely distributed not only centrally but also peripherally. Moreover, it has also been shown to be localized in the gastrointestinal tract, adipose tissue, pancreas, liver, and other organs, and it is hyperactive in metabolic disorders such as obesity, diabetes, dyslipidemia, and liver disease [184,185]. From a physiological point of view, it is involved in numerous functions, such as gastrointestinal motility, anxiety, stress, and nociception, but it also acts through orexigenic signaling, with a role in regulating food intake [43]. The hyperactivation of the ECS leads to an increase in the plasma concentrations of 2-AG and AEA. This hyperactivation is particularly evident in cases of obesity, especially visceral obesity, which is closely related to an increased risk of cardiovascular disease as well as T2DM [43,186].

It is well known that the two endogenous ligands in animal studies regulate hunger by stimulating CB1Rs, contributing to metabolic functioning. ECBs are synthesized and released on demand rather than stored in vesicles. In humans, their levels rise during periods of fasting, and the increase in caloric intake is linked to the administration of AEA and 2-AG in the hypothalamus [41].

Food intake triggers elevated levels of ECBs in the NAc, leading to augmented dopamine (DA) secretion at the central level. By activating CB1Rs in the parabrachial nucleus, ECBs promote food intake and pleasure, leading to increased feeding behavior [25].

Since the ECS plays a role in maintaining energy homeostasis, its dysfunction is linked to certain pathological conditions, such as obesity and diabetes. CB1Rs in the olfactory cortex have been shown in a number of preclinical studies to increase food intake in fasting mice. ECBs facilitated increased aroma detection, followed by increased hunger and food intake with increased caloric levels. During fasting, ECB levels increase, activating CB1Rs at axonal terminals in the olfactory cortex, followed by reduced glutamatergic transmission and granule cell excitation, with odor detection and food intake driven by CB1R activation [187].

A very recent study has shown that CB1R deficiency in non-immune cells favors resistance to diet-induced obesity, whereas, in immune cells, diet-induced obesity is exacerbated. The experimental model was based on the development of bone-marrow-derived chimeric mice lacking CB1Rs. In the non-immune cells of these mice, a deficiency of these receptors was observed, which was associated with the attenuation of obesity.

Instead, CB1R deletion in immune cells caused accelerated weight gain, inflammation, and glucose intolerance [188].

While acute stimulation of CB1Rs induces increased food intake, blocking them with low and moderate doses of antagonists showed hypophagia in both preclinical and clinical studies, with hypophagia induced by the presynaptic suppression of the two neurotransmitters glutamate and GABA. However, when CB1Rs are activated by agonists, DA is released in the neostriatum or prefrontal cortex, and dopaminergic neurons in the midbrain are activated; these neurons mediate the rewarding stimulation [184,189,190].

The claim that prolonged breaks between meals lead to increased levels of ECBs is based on the fact that CB1Rs are distributed in large numbers in the CNS, especially in reward circuits, with their role being to amplify hunger signals by stimulating the desire to feed [147].

The activation of dopaminergic neurons in the VTA will mediate the need for a stimulus, with the ECS controlling the activity of dopaminergic neurons as well as DA signaling [184].

AEA, through the activation of CB1Rs, causes an orexigenic effect, promoting the phenomenon of hyperphagia, which suggests the involvement of ECBs in feeding behavior. This has been shown in preclinical studies by administering AEA to the hypothalamus in the ventromedial nucleus in rats, which generated a hyperphagic effect [147,186].

Both ECBs are synthesized locally by the intestinal tissue, with the 2-AG concentration at this level not being influenced by starvation or re-alimentation, while an increased AEA concentration has been correlated with feeding behavior [147].

Lipid synthesis in the liver is favored by CB1Rs. In wild mice, it was found that feeding them a regular diet resulted in the activation of CB1Rs, an activation characterized by increased fatty acid synthesis, but not in CB1 KO mice [184].

Numerous preclinical and clinical studies have shown that this system is involved in dietary intake and lipogenesis, and CB1R activation can lead to the development of insulin resistance. In controlled clinical trials, the administration of rimonabant, a selective CB1R antagonist, resulted in improved glycemic and lipid profiles, while CB1R agonist therapy was associated with hepatic lipogenolysis in preclinical studies [191].

In obese patients, studies have shown increased plasma levels, suggesting a correlation between adipose tissue and ECB levels, whose elevated plasma value may be due either to adipocytes unable to catabolize ECBs or to activated biosynthesis [192].

In the adipose tissue, both ECBs are present in mature adipocytes, a fact evidenced by increased circulating concentrations of 2-AG in obese individuals [147]. The ECS is also involved in modulating energy storage, a phenomenon observed in animal studies, in which treatment with CB1R antagonists (e.g., rimonabant) led to increased insulin-dependent GLUT-4 glucose transport in adipocytes [147,153,193].

The ECS is involved in hepatic metabolism, as evidenced by the increased expression of ECBs and CB1Rs identified in the livers of mice induced by a high-fat diet, and in CB1R-deficient mice, resistance to obesity induced by a high-fat diet has been observed [193]. CB2Rs are known to be involved in the modulation of immune responses but are also involved in carbohydrate metabolism and body weight modulation. In obese mice, the activation of CB2Rs resulted in insulin resistance and the potentiation of inflammatory markers, and in the case of animals lacking CB2R, an increase in food intake could be observed, but insulin resistance and the risk of developing food-induced obesity decreased [161,194].

It is well known that the main compound in cannabis extracts, Δ^9^-tetrahydrocannabinol (Δ^9^-THC), activates CB1R, resulting in the stimulation of appetite and satiety [43,44].

The following question arises: What is the physiological role of the ECS in pregnancy-associated obesity?

ECS activity can be affected by the diet, as this system is involved in modulating lipogenolysis and fatty acid use in the liver, fat, and muscle tissues. The transgestational effect of maternal obesity has been studied in knockout mice fed a high-lipid diet, and the offspring born later showed higher birth weight and increased blood cholesterol levels compared to offspring from the control group.

The degradation of AEA by FAAH concludes its biological activity. Because CB1Rs play a role in eating, lipid metabolism, and other processes, such as energy expenditure, CB1 knockout (CB1 KO) mice have been found to be resistant to gaining weight on a high-lipid diet [181].

Another study showed that CB1 KO mice eat less food, are lean, and do not develop insulin resistance. In contrast, CB1R activation has a strong orexigenic stimulatory effect [43].

A recent study (2021) based on CRISPR/*Cas9* technology found that in mice lacking CB1Rs in the ventromedial hypothalamus, a lipid-rich diet did not affect body weight, suggesting that these receptors may not be involved in food intake. However, these receptors regulated glucose homeostasis in obese male mice, a model of diet-induced obesity. The regulation of glucose metabolism by these receptors is not dependent on body weight regulation [195].

CBRs are also involved in peripheral effects on energy homeostasis, with energy consumption in the periphery being regulated by the ECS. Numerous studies have shown that weight loss induced by CB1R antagonists (e.g., rimonabant) in rats was possible without being dependent on reduced dietary intake [147].

Peripheral processes such as lipogenesis, stomach emptying, and glucose absorption are regulated by the ECS through CBRs in the adipose tissue, skeletal muscle, and gastrointestinal tract. The metabolic state of the body is constantly monitored by the brain through signals received from peripheral organs, as the control of food intake and energy consumption is coordinated by the ECS, which functions as a communicative circuit between the central and peripheral parts [147].

Clinical studies have also shown the phenomenon of glucose uptake in primary human fat cells, proving the involvement of the ECS. Subcutaneous adipose tissue was taken from two groups of subjects. The first group consisted of five female and four male subjects with an average age of 32, whose body weight was within normal limits. The second group included seven obese females and five obese males with an average age of 34. In both cases, glucose tolerance was found to be normal, but insulin resistance was obvious in subjects in the obese group. The development and expansion of adipose tissue require not only fatty acid intake but also glucose, as assembly involves the translocation of glucose transporter type 4 (GLUT4), a step dependent on insulin and glucose transport [153].

Several processes, including inflammation and phenomena typical of cardiac diseases, including acute heart attack, atherosclerosis, and others, have been linked to ECS involvement in cardiovascular disease. At the cardiac level, in the case of ischemia, studies have shown that in both animals and humans, CBRs, together with their endogenous ligands, are upregulated [196].

It appears that most of the effects of CB1R on the cardiovascular system may be due to the inhibition of the parasympathetic autonomic nervous system and the activation of the sympathetic nervous system [197].

In mouse and human atherosclerotic plaques, CB2R expression was significantly increased. The clinical part of the study was conducted on 24 patients aged 63–85 from whom atheroma plaques were removed from carotid areas. Carotid artery samples from animal (mouse) models were also taken. In these plaques, CB2R expression was significantly increased, but no differences in the levels of these genes were observed between stable and vulnerable plaques [198].

The protective role of CB2Rs in cardiovascular disease has been highlighted by a number of recent studies, with the expression of these receptors observed in cardiomyocytes, human atherosclerotic plaques, and macrophages. Cardioprotective action through CB2R activation has been demonstrated in both preclinical and clinical studies, with evidence of action on ischemic lesions, reducing the rate of myocardial infarction and ventricular arrhythmias [199].

### 5.2. Cannabinoid Receptor Modulators in Obesity

In researchers’ attempts to find new targets for the treatment of obesity, the development of antagonists/inverse agonists of CB1Rs still represents a promising therapeutic option, even after the experience of withdrawing rimonabant from the market. In the research, design, and optimization of these molecules, previous studies showed that besides the lack of penetration through the blood–brain barrier to avoid central adverse reactions, selectivity for CB1Rs over CB2Rs plays an important role: CB2R agonists have been found to have cardioprotective effects, so blocking these receptors could worsen certain cardiometabolic conditions [182,200].

Rimonabant is the first potent and selective CB1R antagonist/inverse agonist developed in 1994 [201], exhibiting both centrally and peripherally mediated metabolic effects [154]. Rimonabant was first approved as an anti-obesity medication in Europe in 2006, being indicated for obese and overweight patients with major metabolic comorbidities [202]. After its withdrawal, the molecule still remained a model in ECS research due to its involvement in obesity and other metabolic diseases, as well as for the development of compounds with an improved pharmacological profile [154].

In preclinical studies, rimonabant treatment led to decreased food intake and body weight loss in diet-induced obese and genetically obese mice, effects that were not observed in CB1 knockout mice, proving that they are mediated particularly by CB1Rs [202]. The study by Herling et al. demonstrated the ability of rimonabant to induce lipolysis, with immediate increases in fat oxidation and free fatty acid levels, after the first administration in postprandially challenged rats. According to these findings, rimonabant’s effects on lipolysis and body weight reduction are not a result of decreased food intake but rather an effect of the drug itself [203]. Gary-Bobo et al. found that rimonabant improved metabolic syndrome features, such as inflammation and dyslipidemia, as well as hepato-steatosis associated with obesity. Thus, daily treatment with rimonabant for 8 weeks in obese (fa/fa) rats reduced hepatomegaly; the plasma levels of alanine aminotransferase, gamma-glutamyl transferase (γ-GT), and alkaline phosphatase; and the elevated levels of TNF-α associated with steatohepatitis. The triglyceride, free fatty acid, and total cholesterol levels in the plasma were reduced, and the HDL/LDL ratio was raised, all of which indicated an improvement in the lipid profile and dyslipidemia [204].

Recent research showed that when DIO mice were fed a high-fat diet and then treated with rimonabant, they lost a significant amount of fat and weight. Additionally, rimonabant has been shown to improve metabolism and decrease inflammation in adipose tissue [205]. The compound was shown in another study to prevent weight gain and restore insulin tolerance in mice that had been fed a high-fat diet. In addition, high-voltage-activated Ca^2+^ channel function and the Cav1.1 expression level in skeletal muscle cells were both found to be restored by rimonabant treatment after being diminished by a high-fat diet. One of the drug’s purported protective effects on diet-induced obesity is to maintain a healthy balance between the body’s appetite and glucose homeostasis, according to the study’s authors [63,206].

The therapeutic potential against obesity identified in preclinical studies of rimonabant led to the initiation and performance of clinical trials, with some of them being terminated before completion due to the withdrawal of the drug from the market in 2008 [63]. A selection of the main clinical trials conducted with rimonabant, as well as the main effects obtained on obesity and lipid metabolism, is given in Table 3. Between 2001 and 2004, the Rimonabant in Obesity (RIO) trials were among the earliest and largest clinical trials of rimonabant’s efficacy.

Patients who were overweight or obese and had certain comorbidities without diabetes [162,207,208] or with T2DM [163] were included in these trials, which were randomized, double-blind, placebo-controlled, multicenter comparisons of rimonabant efficacy to the placebo.

The results from the four RIO clinical trials were found to be consistent, according to an analysis by Scheen et al. After 12 months of follow-up, in all three RIO studies, non-diabetic patients in the rimonabant 20 mg treatment group combined with a hypocaloric diet lost significantly more weight (−4.7 to −5.4 kg) and experienced a greater decrease in waist circumference (−3.6 to −4.7 cm) than those in the placebo group. Reductions in triglycerides (−12.4% to −15.1%) and increases in HDL cholesterol (+7.2% to +8.9%) (results expressed as placebo-subtracted) were also observed in the same groups, indicating significant improvements in cardiometabolic risk factors [209]. There was a positive correlation between changes in plasma adiponectin levels and changes in HDL cholesterol levels (r = 0.27, *p* < 0.001) [162] in the RIO-Lipids study, with adiponectin levels increasing by 57.7% in the rimonabant 20 mg dose group. Metabolic dysfunction, obesity, insulin resistance, diabetes, and cancer have all been linked to low levels of the adipose tissue hormone adiponectin [210]. Additionally, plasma leptin levels (*p* < 0.001) and C-reactive protein levels (*p* = 0.020) were found to be significantly reduced [162]. The prevalence of metabolic syndrome was found to be reduced in all three RIO studies involving non-diabetic patients [209]. Patients with T2DM who were overweight or obese and who had inadequate control despite treatment with metformin or sulfonylureas alone were included in the RIO-Diabetes study. Rimonabant 20 mg combined with a mildly hypocaloric diet and exercise recommendation led to significant weight and waist circumference reductions after one year in comparison to the placebo. Glycated hemoglobin (HbA1c), HDL, and triglyceride levels all improved with rimonabant 20 mg treatment compared to the placebo, as well as improvements in fasting glucose levels, homeostasis model assessment of insulin resistance (HOMA-IR), and the prevalence of metabolic syndrome. In addition to the previously mentioned improvements in cardiometabolic factors, the rimonabant 20 mg group also showed a decrease in alanine aminotransferase (ALT), which is typically elevated in hepatic steatosis [163,209].

The beneficial results for obesity are supported by a meta-analysis that included the four RIO studies. This study adds to the evidence that rimonabant doses of 20 mg/day can increase the chance of psychiatric side effects. More than twice as many rimonabant-treated patients dropped out of treatment because of depressive mood disorders as placebo-treated patients [211]. Common side effects in the four studies included depressive disorders, anxiety, irritability, nausea, nasopharyngitis, dizziness, arthralgia, headache, etc., some of them leading to treatment discontinuation [162,163,207,208].

Pan et al. compared the positive effects of a hypocaloric diet combined with rimonabant 20 mg/day or a placebo in an Asian population to those seen in studies conducted in Europe and the US over a 9-month period. The study included 643 patients and found that rimonabant at 20 mg/day resulted in significant weight loss (−2.99 kg, *p* < 0.0001) and waist circumference reductions compared to the placebo. In addition, the HDL cholesterol and triglyceride levels of the rimonabant-treated group significantly improved compared to the placebo group. Rimonabant at a daily dose of 20 mg was well tolerated in the study population, which reflects the drug’s favorable safety profile. Negative reactions such as dizziness, palpitations, diarrhea, nausea, respiratory tract infections, insomnia, depression, and anxiety were the most common. The percentage of patients who discontinued treatment due to adverse events was similar in the two treatment groups (3.1% versus 2.8% in the rimonabant and placebo groups, respectively). However, among psychiatric disorders, depression and anxiety were more frequent in the group treated with rimonabant compared to the placebo group (4.1% and 3.8%, respectively, compared to 0.9% and 1.5%, respectively) [212].

**Table 3:** Main clinical trials of rimonabant in the treatment of obesity.

Study Type	Subjects	Duration	Drug Dose (Oral)	Main Effects on Obesity and Lipid/Glucose Metabolism (Compared to Placebo)	Reference
Double-blind, placebo-controlled, multicenter	Overweight or obese adults (BMI 27–40 kg/m^2^) with untreated dyslipidemia and without DM (*n* = 1036)	1 year	5 or 20 mg/day	Weight loss, reduction in waist circumference, increase in HDL cholesterol, reduction in triglyceride levels, increase in plasma adiponectin levels, and decrease in plasma leptin levels	Després et al. [162]
Double-blind, placebo-controlled, multicenter	Adults with BMI ≥ 30 kg/m^2^ or ≥27 kg/m^2^ with untreated dyslipidemia, hypertension, or both (*n* = 1507)	1 year	5 or 20 mg/day	Weight loss, reduction in waist circumference, increase in HDL cholesterol, reduction in triglyceride levels, improvements in insulin resistance, and prevalence of the metabolic syndrome	Van Gaal et al. [208]
Double-blind, placebo-controlled, multicenter	Obese adults (BMI ≥ 30 kg/m^2^) or overweight adults (BMI ≥ 27 kg/m^2^) with treated or untreated hypertension or dyslipidemia (*n* = 3045)	2 years	5 or 20 mg/day	Weight loss, reduction in waist circumference, reduction in triglyceride levels, increase in HDL cholesterol, and decrease in fasting insulin levels	Pi-Sunyer et al. [207]
Double-blind, placebo-controlled, multicenter	Overweight or obese (BMI 27–40 kg/m^2^) T2DM adults (*n* = 1047)	1 year	5 or 20 mg/day	Weight loss, reduction in waist circumference, reduction in hemoglobin A1c levels, and improvements in fasting glucose concentrations and HOMA-IR, HDL cholesterol, triglyceride, and non-HDL cholesterol concentrations; decreased appetite, ease in following the diet, and less desire for high-fat foods and sweets	Scheen et al. [163]
Double-blind, placebo-controlled, multicenter	Adults with BMI ≥ 30 m^2^ or ≥27 kg/m^2^ with treated/untreated dyslipidemia, hypertension, or both (*n* = 1507)	2 years	5 or 20 mg/day	Weight loss, reduction in waist circumference, increase in HDL cholesterol, reduction in triglyceride levels, and improvements in fasting glucose and insulin levels, insulin resistance, and metabolic syndrome prevalence	Van Gaal et al. [213]
Double-blind, placebo-controlled, multicenter	Adults with a waist circumference of 102 cm (men)/88 cm (women) with atherogenic dyslipidemia (*n* = 803)	1 year	20 mg/day	Weight loss, reduction in waist circumference, increase in HDL cholesterol, reduction in triglyceride levels, decrease in subcutaneous and visceral adipose tissue, reduction in liver lipid content, improvement in several cardiometabolic risk markers, and decrease in fasting glucose and insulin levels	Després et al. [214]
Double-blind, placebo-controlled, multicenter	Adults (age > 55 years) with a waist circumference of 102 cm (men)/88 cm (women) and cardiovascular comorbidity or at least two major cardiovascular risk factors (*n* = 18,695)	13.8 months (mean follow-up)	20 mg/day	Discontinued: cessation of all rimonabant trials in November 2008	Topol et al. [215]
Double-blind, placebo-controlled, multicenter	Adults with BMI ≥ 25 kg/m^2^ without diabetes (*n* = 643)	9 months	20 mg/day	Weight loss, reduction in waist circumference, increase in HDL cholesterol, and reduction in triglyceride levels	Pan et al. [212]
Randomized, placebo-controlled	Obese (BMI 30–35 kg/m^2^) Caucasian postmenopausal women (*n* = 30)	12 weeks	20 mg/day	Similar results for weight loss and waist circumference reduction and increase in lipolysis and fatty acid oxidation	Backhouse et al. [216]
Open-label	Obese adults (BMI ≥ 30 kg/m^2^) with T2DM (*n* = 20)	6 months	20 mg/day	Weight loss, reduction in waist circumference, reduction in hemoglobin A1c levels, and increase in HDL cholesterol	Heppenstall et al. [217]
Double-blind, placebo-controlled, multicenter	Obese adults (BMI 30–45 kg/m^2^) with binge eating disorders (*n* = 289)	6 months	20 mg/day	Weight loss, reduction in waist circumference, and reduction in binge eating scale total score	Pataky et al. [218]
Double-blind, placebo-controlled	Adults (age between 35 and 70 years) with metabolic syndrome (*n* = 37)	48 weeks	20 mg/day	Weight loss, decrease in liver fat, decrease in intra-abdominal fat, and decrease in ALT, gamma-GT, triglycerides, fasting plasma glucose, fasting plasma insulin, and HOMA-IR	Bergholm et al. [219]

Legend: BMI: body mass index; HDL: high-density lipoprotein; HOMA-IR: homeostasis model assessment of insulin resistance; T2DM: type 2 diabetes mellitus.

Taranabant, like rimonabant, is another centrally acting CB1R antagonist/inverse agonist that has been investigated for use in the treatment of obesity. It has been shown to significantly reduce weight and food intake in diet-induced obese mice in preclinical studies, but these effects have not been seen in CB1R knockout mice [63]. Research comparing rimonabant and taranabant found that the former led to greater weight loss in obese mice than in lean mice, while the latter led to similar weight loss in both groups [220]. 

The main clinical trials of taranabant are shown in Table 4. In larger studies, different doses of taranabant have shown beneficial effects in patients with obesity, leading to weight loss, a reduction in waist circumference [221], and improvements in cardiometabolic risk factors (increase in HDL cholesterol, reduction in triglyceride levels, improvements in insulin sensitivity [222], reduction in HbA1c levels, or reduction in fasting plasma glucose) [223]. Despite clinically significant results for obesity, the toxicological profile of taranabant has led to the discontinuation of the clinical development of the substance. In single-dose studies, taranabant was generally well tolerated, and no serious adverse events were reported [224]. In studies with the daily dosing of taranabant over 1 or 2 years, a higher number of overall drug-related adverse events were observed in the taranabant-treated groups compared to the placebo groups. Among the most common adverse reactions in the taranabant-treated groups compared with placebo-treated groups were gastrointestinal disorders (diarrhea, nausea, and vomiting), nervous system disorders (dizziness and headache), psychiatric disorders (including depression and anxiety), and irritability [221,222,223,225].

Ibipinabant is another first-generation antagonist/inverse agonist of CB1Rs; it appears to be as effective as rimonabant in decreasing the intake of a highly palatable diet in Wistar rats, but with a lower degree of occupancy of brain CB1Rs [228], suggesting that part of the anti-obesity effect of CB1R antagonists/inverse agonists is due to their action on peripheral receptors [229]. Ibipinabant has been included in Phase II (NCT00388609) and Phase III (NCT00541567) clinical trials to study its efficacy in obese or overweight patients with comorbidities and obese or overweight T2DM patients, respectively. The trials were terminated and withdrawn due to rimonabant’s central nervous system safety concerns [229].

Otenabant, another brain-penetrant CB1R antagonist/inverse agonist, is a purine derivative developed by Pfizer. Hadcock et al. observed that otenabant displays a high affinity for human CB1Rs. In their pharmacological evaluation of the compound, they demonstrated its efficacy in decreasing food intake in a dose-dependent manner in rats, in addition to increasing energy expenditure and fat oxidation. Given orally at a dose of 10 mg/kg for 10 days, the compound decreased body weight in diet-induced obese mice by 9% (when compared to vehicle-treated animals) [230]. The long-term efficacy and safety of otenabant were studied in three randomized, double-blind, placebo-controlled, multicenter, Phase III clinical trials in patients who were either overweight or obese and had or did not have T2DM. Due to shifting views on the safety profile of CB1R-related drugs and the presumed difficulty in gaining regulatory approval, the sponsor company decided to discontinue all three otenabant trials in 2008 [231]. Most patients enrolled in the three studies lasted the full year, and those who took otenabant lost significantly more weight and inches around their middles compared to the placebo in every population studied. Diarrhea, nausea, nasopharyngitis, and headache were the most common adverse reactions in the otenabant-treated groups compared to placebo groups. In addition, in all three trials, the otenabant-treated groups had a higher rate of psychological and psychiatric symptoms than the placebo groups [231].

Another representative of first-generation CB1R antagonists/inverse agonists studied for its potential against obesity is surinabant [229], a structural analog of rimonabant [232]. Rinaldi-Carmona et al. showed that surinabant can reduce ethanol or sucrose consumption and food intake in rats and mice, as well as dose-dependently decrease food consumption in both fed and fasted rats [201]. Surinabant has been investigated as a potential treatment for various addictions, such as smoking and alcoholism [233], and was included in a Phase II study (NCT00239174) evaluating the effect of the drug on weight loss, as well as its safety and tolerability, when prescribed in addition to a hypocaloric diet in obese patients. No published results are available for this study.

The therapeutic potential of CB1R blockade for obesity and its complications has prompted a keen interest among researchers to design and develop compounds that act on peripheral CB1Rs, maintaining the beneficial cardiometabolic and anti-obesity effects and avoiding central-nervous-system-mediated adverse reactions.

TM38837 is among the first CB1R antagonists/inverse agonists developed that act peripherally, showing a brain/plasma ratio of 1:33 [182,234], with similar effects to rimonabant in inducing weight loss in obese mice [235]. Preclinical PET study results in nonhuman primates show a significantly lower tendency of TM38887 to penetrate the brain compared to rimonabant at an expected clinical therapeutic dose of 20 mg [236]. Klumpers et al. studied the central and peripheral effects of TM38837 in healthy human subjects. The absence of central adverse reactions at a dose of 100 mg suggests that TM38837 has very little penetration across the blood–brain barrier at this dose [237]. A more recent study in mice showed that TM38837 caused fear-promoting side effects only after the administration of the highest dose tested, 100 mg/kg, which is 10 times higher than the rimonabant dose that produced similar effects [235].

JD5037 is another peripherally restricted CB1R antagonist/inverse agonist analog of the first-generation brain-penetrating compound ibipinabant. JD5037 exhibits low brain penetrance, a high affinity for CB1R, and >700-fold selectivity for CB1R over CB2R. At a dose of 3 mg/kg/day for 28 days, JD5037 elicited similar results to ibipinabant, its parent compound, in reducing food intake, body weight, and fat mass in diet-induced obese mice. Likewise, the authors suggest that the hypophagic and weight-reducing effects of JD5037 are leptin-dependent, with the drug producing and restoring endogenous leptin sensitivity in diet-induced obese mice by reversing hyperleptinemia [238]. JD5037 also seems to improve several cardiometabolic factors in diet-induced obese mice, such as insulin resistance, plasma triglyceride levels, and LDL levels, as well as reducing liver inflammation and lipogenesis and increasing fatty acid oxidation [200,238].

BPR0912 is a CB1R-selective antagonist that possesses minimal blood–brain barrier permeability. Body weight and fat mass were reduced, and serum levels of leptin, insulin, and triglycerides, as well as hepatic steatosis, were all reduced in BPR0912-treated diet-induced obese mice. Serum glucose and free fatty acid levels also changed slightly [239].

TXX-522 was discovered to be a peripheral CB1R-selective antagonist that warrants further study. Only about 2% of TXX-522 was detected in brain tissue, indicating poor BBB penetrance, whereas over 60% of rimonabant was distributed in the brain under the same conditions [176]. Furthermore, TXX-522 did not alter the food consumption of either normal or diet-induced obese mice, indicating a lack of activity on CB1Rs in the brain (the hypothalamus and mesolimbic regions appear to be involved in appetite control) [240]. In diet-induced obese mice, TXX-522 reduced body weight and abdominal fat to the same extent as rimonabant [176].

More recently, Han et al. developed two new peripherally restricted CB1R antagonists/inverse agonists, AJ5012 and AJ5018, by making some modifications to the structure of rimonabant [178]. Both compounds showed brain/plasma concentration ratios that were much lower than those of rimonabant [178,241]. Diet-induced obese mice treated with AJ5012 showed improved metabolic profiles and decreased adipose tissue inflammation. In addition, although no changes were observed in food intake in AJ5012-treated diet-induced obese mice, the drug produced decreases in body weight and adiposity, but to a lesser extent than rimonabant, probably due to acting only on energy expenditure [178]. Similarly, the efficacy of AJ5018 was studied in comparison with rimonabant in rodent models of obesity. AJ5018 reduced food intake and body weight, albeit to a lesser extent than rimonabant, when compared to the vehicle control group. The analysis of body mass composition revealed that the weight loss was attributable solely to a decrease in total fat mass, with no corresponding change in lean body mass. In diet-induced obese mice, both compounds improved hepatosteatosis and hepatocellular damage, decreased plasma leptin levels, enhanced lipid and carbohydrate profiles, and reduced adipose tissue inflammation [178].

MRI-1867 is another CB1R antagonist/inverse agonist with limited brain penetrance that also inhibits iNOS. DIO mice treated with MRI-1867 lost weight, decreased their food intake, and increased their energy expenditure and fat oxidation, demonstrating the anti-obesity efficacy of the new hybrid inhibitor [242]. Important antifibrotic effects of MRI-1867 were also observed in the liver [243], and it was found to reduce obesity-related renal injury, inflammation, fibrosis, and oxidative stress in the kidneys [242]. This compound is promising for further clinical development because it inhibits peripheral CB1R and iNOS simultaneously and is orally bioavailable [242].

Several other peripherally restricted CB1R antagonists/inverse agonists were studied and showed anti-obesity effects and improvements in hormonal or metabolic abnormalities in rodent models of obesity, for example, URB 447, MJ08, SR141716, AM6545, ENP11, and NESS06SM [63,154,229], all of them requiring further investigation. 

Research in recent years has expanded our knowledge of the endocannabinoid system and its implications for obesity [25]. Thus, CB1R blockade by compounds with a good pharmacotoxicological profile represents a promising future therapeutic target in the treatment of obesity and related complications.

## 6. Conclusions

Some of the mechanisms involved in the ECS’s complex network of signaling molecules and receptors are only partially understood. The functions of the ECS under a wide range of conditions, such as diabetes, obesity, and cardiovascular diseases, in addition to its role in inflammation and immunomodulation, are explored in this review. 

The two metabotropic receptors CB1R and CB2R, with structural similarities to GPCRs, play a crucial role in energy metabolism and are expressed in nearly all mammalian tissues. They mediate distinct physiological effects through signaling pathways involved in a wide range of processes.

Modulating CBRs holds therapeutic promise for improving various pathological conditions, from CNS disorders to cardiovascular and metabolic disorders such as obesity/metabolic syndrome. However, further testing is necessary to ensure the tolerability and safety of CBR modulators before clinical trials.

By gaining a more in-depth understanding of the molecular biology and pharmacology of CBRs, it will be possible to develop new medications that are more selective in their targeting of these receptors, making them both safer and more effective.

## Figures and Tables

**Table 1:** The effects of CBR modulators in inflammation and immunomodulation.

Compound	Effect	Disease Model and Species	Reference
CBD	Induced CB2R inverse agonism at concentrations below those at which it displaced [^3^H] CP55940 from these receptors	hCB2-CHO cell membranes MF1 mice C57BL/6 CB1 receptor knockout mice	Thomas et al. [88]
SR144528 and AM630	Inhibited IL-6-induced IgM production Protein kinase STAT3 pathway Transcription factors such as Bcl-6 and Pax5	Human SKW 6.4 cells	Feng et al. [94]
SR144528	Counteracted CP 55,940’s inhibition of forskolin-stimulated AC activity	CHO-CB1 cells expressing CB2Rs	Rinaldi-Carmona et al. [95]
	Selectively inhibited CP 55,940-induced p22/p44 MAPK activity	Cells expressing CB2Rs	Cabral et al. [91]
	Increased forskolin-stimulated cAMP accumulation		Kumar et al. [96]
JTE-907	Proved no effect on the development of T-cell subtypes such as Th1, Th2, Th9, or Th17 Activated the p38 MAPK pathway	Mouse model of IBD	Gentili et al. [97]
JTE-907 and SR144528	Prevented the inflammatory response	Mouse model of allergic dermatitis after topical treatment with DNFB	Ueda et al. [100]
SMM-189	Inhibited microglia polarization toward the proinflammatory M1 phenotype and the pro-healing M2 phenotype	2,6-Dihydroxy-biphenyl-arylmethanone scaffold	Alghamdi et al.
	Suppressed the expression of inflammatory cytokines and chemokines	Primary microglia derived from humans and mice	Presley et al. [102] Reiner et al. [103]
	Reduced inflammatory responses	Classically activated microglia	Yu et al. [87]
	Prevented neuronal damage against glutamate excitotoxicity	Primary cultures of brain cells isolated from rat hippocampal tissues	
	Partially decreased inflammatory markers Diminished the severity of comorbid behavioral deficiencies	Mouse model of status epilepticus induced by kainite	
	Reduced the increased level of COX-2 in the hippocampus and cerebral cortex Reduced injury and functional deficits after prolonged seizures		Buisseret et al. [104]
Sch. 414319	Reduced clinical symptoms of EAE Suppressed cell migration in vivo 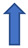 Controlled L-plastin phosphorylation	Lewis rat strain	Lunn et al. [84]
Sch.336	Blocked CB2-expressing recombinant cells from moving toward the CBR agonist 2-AG	In vitro model of recombinant cells	Lunn et al. [105]
	Modified leukocyte migration in the presence of the CBR agonist HU-210 Modified chemokine-induced cell recruitment into a CCL2-soaked gel foam sponge	In vivo experiments	
AM630	Inhibited the expression of CB2R	RAW 264.7 cells	Geng et al. [107]
	Reduced inflammatory osteolysis	Titanium particles generated due to prosthesis wear	
	Significantly decreased the protein expression of specific proinflammatory cytokines, i.e., IL-1β and TNF-α	RAW cells cultured with titanium (Ti) particles	
	Activated the influx of Ca^2+^ into sensory TG neurons → pharmacological cross-desensitization of the TRPV1 channel		
	Activated TRPA1 when co-expressed with TRPV1		Patil et al. [111]
	Inhibited capsaicin-, WIN55,212-5-, and mustard-oil-induced responses	Cultured TG neurons	
SMM-189	Decreased neuronal death in the cortex, striatum, and amygdala Restoration of normal oscillatory activity in the hippocampus and the prefrontal cortex	Mouse model of TBI	Jordan et al. [113]

Legend: DNFB: dinitrofluorobenzene; EAE: experimental autoimmune encephalomyelitis; IBD: irritable bowel disease; IL-1β: interleukin-1β; TBI: traumatic brain injury; TG: trigeminal; TNF-α: tumor necrosis factor-α; TRPV1: transient receptor potential vanilloid 1; TRPA1: transient receptor potential ankyrin 1; ↑ direct connection between mechanism and effect.

**Table 2:** The effects of CBR modulators in DM.

Compound	Effect	Disease Model and Species	Reference
AM251	Beneficial effect on glucose concentrations	Young obese diabetic mice	Irwin et al. [167]
	Improved insulin sensitivity		
	Promoted insulin secretion	Isolated human islets	Ruz-Maldonado et al. [168]
	Prevented apoptosis in islets	Isolated mouse and human islets	
AM6545 and AM4113	Reduced insulin levels and resistance to near-control values Restoration of adiponectin levels Reduction in TNF-α Prevented the development of insulin resistance	High-fructose- and high-salt-diet-fed Wistar rats	Eid et al. [171]
	Restoration of adiponectin levels	Metabolic syndrome rats	
JD5037	Improved renal inflammation	STZ-induced diabetic mice	Hinden et al. [175]
	Normalized impaired kidney function		
	Improved tubular injury		
	Normalized collagen-3, collagen-1, and fibronectin-1 levels		
TXX-522	Reduced fasting blood glucose	Male Sprague-Dawley rats	Chen et al. [176]
	Improved glucose intolerance		
(S)-MRI-1867	Improved glycemic control Lowered leptin levels	Obese mice	Roger et al. [177]
AJ5012	Improved insulin sensitivity and glycemic control Suppressed adipose tissue inflammation via NLRP3 inflammasome	Diet-induced obese mice and db/db mice	Han et al. [178]
LH-21	Increased insulin sensitivity	Genetic ZDF rats and diet-induced obese rodent models	Dong et al. [179]
	Cytoprotective effects on pancreatic islets Reduced diabetes risk factors (e.g., glucose handling, tissue inflammation)	Mouse model of obesity and pre-diabetes	Romero-Zerbo et al. [180]

Legend: NLRP3: nucleotide-binding domain and leucine-rich repeat protein 3; ZDF: Zucker diabetic fatty.

**Table 4:** Main clinical trials of taranabant in the treatment of obesity.

Study Type	Subjects	Duration	Drug Dose (Oral)	Main Effects on Obesity and Lipid/Glucose Metabolism (Compared to Placebo)	Reference
“Dose Range-Finding Weight-Loss Study” Double-blind, placebo-controlled, multicenter	Adults with a BMI ≥ 30 kg/m^2^ and ≤43 kg/m^2^ with no significant comorbidities (*n* = 533)	12 weeks	0.5, 2, 4, or 6 mg	Weight loss and reduction in waist circumference in a dose-dependent manner	Addy et al. [224]
“24-h Food Intake Study” Double-blind, placebo- and active-controlled, single-dose, four-period crossover, single-center	Overweight and moderately obese (BMI 25–35 kg/m^2^) male adults (*n* = 36)	Acute	4 or 12 mg	Reduction in total caloric intake (postdose meal)	
“Resting Energy Expenditure Study” Double-blind, placebo- and active-controlled, single-dose, four-period crossover, single-center	Overweight and moderately obese (BMI 25–35 kg/m^2^) male adults (*n* = 17)	Acute	4 or 12 mg	Increase in fat metabolism	
Double-blind, placebo-controlled, single-rising-oral-dose, single-center	Healthy male adults (*n* = 24)	Acute	0.5–600 mg	No significant changes from baseline in appetite/satiety	Addy et al. [226]
Double-blind, placebo-controlled, double-center	Healthy male adults (*n* = 60)	24 days	5, 7.5, 10, 25 mg/day	No consistent changes from baseline in the appetite/satiety visual analog scale questionnaire	Addy et al. [227]
Double-blind, placebo-controlled, multicenter	Obese adults with a BMI ≥ 30 kg/m^2^ and ≤43 kg/m^2^ or BMI ≥ 27 kg/m^2^ and ≤30 kg/m^2^ with comorbidities (*n* = 2502)	2 years	2, 4, 6 mg/day	Weight loss, reduction in waist circumference and body fat percentage, increase in HDL cholesterol, reduction in triglyceride levels, improvements in insulin sensitivity, and increase in plasma adiponectin levels	Aronne et al. [222]
Double-blind, placebo-controlled, multicenter	Obese adults (BMI 27–43 kg/m^2^) with T2DM (*n* = 623)	1 year	0.5, 1, 2 mg/day	Weight loss, reduction in waist circumference, reduction in triglyceride levels, reduction in HbA1c levels, reduction in fasting plasma glucose, and improvements in insulin sensitivity	Kipnes et al. [223]
Double-blind, placebo-controlled, multicenter	Obese adults with a BMI ≥ 30 kg/m^2^ and ≤43 kg/m^2^ or BMI ≥ 27 kg/m^2^ and ≤30 kg/m^2^ with comorbidities (*n* = 1041)	1 year	0.5, 1, 2 mg/day	Weight loss, reduction in waist circumference, decreases in high-sensitivity C-reactive protein, increase in plasma adiponectin levels, and no significant improvement in fasting serum insulin, insulin sensitivity, or fasting plasma glucose	Proietto et al. [221]
Double-blind, placebo-controlled, multicenter	Obese adults (BMI 30–43 kg/m^2^) (*n* = 784)	1 year	0.5, 1, 2 mg/day	Weight loss, reduction in waist circumference, increase in HDL cholesterol, no significant changes in plasma adiponectin, decrease in C-reactive protein, and decrease in triglyceride levels	Wadden et al. [225]

Legend: BMI: body mass index; T2DM: type 2 diabetes mellitus.

## Data Availability

No new data were created or analyzed in this study. Data sharing is not applicable to this article.

## Abbreviations

AA	Arachidonic acid
2-AG	2-Arachidonoylglycerol
AC	Adenylate cyclase
AEA	Anandamide
ALT	Alanine aminotransferase
ATM	Adipose tissue macrophage
BBB	Blood–brain barrier
BMI	Body mass index
cAMP	Cyclic adenosine monophosphate
CB1R	Cannabinoid-receptor type 1
CB2R	Cannabinoid-receptor type 2
CBD	Cannabidiol
CBR	Cannabinoid receptor
CNS	Central nervous system
CRP	C-reactive protein
DA	Dopamine
DALY	Disability-adjusted life-years
DM	Diabetes mellitus
DN	Diabetic nephropathy
DPN	Diabetic peripheral neuropathy
DRP	Diabetic retinopathy
EAE	Experimental autoimmune encephalomyelitis
ECB	Endocannabinoid
ECS	Endocannabinoid system
ERK	Extracellular signal-regulated kinase
FAAH	Fatty acid amide hydrolase
FDA	Food and Drug Administration
FR	Fixed-ratio
GLUT 4	Glucose transporter type 4
GPCR	G-protein-coupled receptor
GSIS	Glucose-stimulated insulin secretion
HbA1C	Glycated hemoglobin
HDL	High-density lipoprotein
HOMA-IR	Homeostasis model assessment of insulin resistance
IDF	International Diabetes Federation
IKK-β	Inhibitor of nuclear factor kappa-B kinase subunit β
iNOS	Inducible NO synthase
IRF5	Interferon regulatory factor-5
JNK	c-Jun N-terminal kinase
MAPK	Mitogen-activated protein kinase
MEK	Mitogen-activated protein kinase kinase
MNU	N-Methyl-N-nitrosurea
NAc	Nucleus accumbens
nAChR	Nicotinic acetylcholine receptor
NCD	Non-communicable disease
NK	Natural killer
NLRP3	Nucleotide-binding domain and leucine-rich repeat protein 3
NSAID	Non-steroidal anti-inflammatory drug
PI3K	Phosphatidylinositol 3-kinase
PPAR	Peroxisome proliferator-activated receptor
PR	Progressive ratio
PUFA	Polyunsaturated fatty acid
R	Receptor
ROS	Reactive oxygen species
SC	Synthetic cannabinoid
sP	Sardinian alcohol-preferring
STRATUS	Studies with Rimonabant and Tobacco Use
STZ	Streptozotocin
T1DM	Type 1 diabetes mellitus
T2DM	Type 2 diabetes mellitus
TNF-α	Tumor necrosis factor-α
TRPA1	Transient receptor potential ankyrin 1
TRPV1	Transient receptor potential vanilloid 1
US	United States
VTA	Ventral tegmental area
WHO	World Health Organization
ZDF	Zucker diabetic fatty
γ-GT	Gamma-glutamyl transferase
∆^8^-THCV	∆^8^-Tetrahydrocannabivarin
Δ^9^-THC	Δ^9^-Tetrahydrocannabinol
AA	Arachidonic acid
